# Body-size increase in crinoids following the end-Devonian mass extinction

**DOI:** 10.1038/s41598-018-27986-x

**Published:** 2018-06-25

**Authors:** Krzysztof R. Brom, Mariusz A. Salamon, Przemysław Gorzelak

**Affiliations:** 1 University of Silesia in Katowice, Faculty of Earth Sciences, Department of Palaeontology and Stratigraphy, Sosnowiec, Poland; 2 WNoZ UŚ - Centre for Polar Studies KNOW (Leading National Research Centre), Katowice, Poland; 3 Institute of Paleobiology, Polish Academy of Sciences, Warsaw, Poland

## Abstract

The Devonian period ended with one of the largest mass extinctions in the Earth history. It comprised a series of separate events, which eliminated many marine species and led to long-term post-extinction reduction in body size in some groups. Surprisingly, crinoids were largely unaffected by these extinction events in terms of diversity. To date, however, no study examined the long-term body-size trends of crinoids over this crucial time interval. Here we compiled the first comprehensive data sets of sizes of calyces for 262 crinoid genera from the Frasnian-Visean. We found that crinoids have not experienced long-term reduction in body size after the so-called Hangenberg event. Instead, size distributions of calyces show temporal heterogeneity in the variance, with an increase in both the mean and maximum biovolumes between the Famennian and Tournaisian. The minimum biovolume, in turn, has remained constant over the study interval. Thus, the observed pattern seems to fit a Brownian motion-like diffusion model. Intriguingly, the same model has been recently invoked to explain morphologic diversification within the eucladid subclade during the Devonian-early Carboniferous. We suggest that the complex interplay between abiotic and biotic factors (i.e., expansion of carbonate ramps and increased primary productivity, in conjunction with predatory release after extinction of Devonian-style durophagous fishes) might have been involved not only in the early Mississippian diversity peak of crinoids, but possibly also in their overall passive expansion into larger body-size niches.

## Introduction

Body size is a key biological property of organisms, which has a significant influence on life functions, generation time, population and home range sizes^1^. Numerous works reporting the changes in body size of different groups at different length scales have been published^2–5^. Recent global-data studies suggest that animals generally increased their sizes over Phanerozoic^4^. An increase in body size over evolutionary time, a pattern commonly referred to as “Cope-Depéret” rule, is thought to confer many advantages upon organisms, but also induces costs and problems^6^. Indeed, counter-examples documenting reduction of body sizes are also known^7–11^. Notably, one of the most intriguing evolutionary phenomenon is the Lilliput effect^11^, which refers to a decrease in body size of fauna associated with the aftermath of extinctions. In general, four models were invoked to explain this effect: extinction of large taxa, post-crisis appearance of many small taxa, temporary disappearance of large taxa and within-lineage size decrease^8^.

The Late Devonian extinction is typically considered to be one of the Big Five mass extinctions. However, this extinction was not geologically instantaneous, in that it is characterized by a series of extinction pulses associated with anoxic events^12–14^. Furthermore, as stressed by Stigall^15^ diversity decline throughout the Late Devonian was mostly caused by a reduction in origination rates rather than elevated extinction. At around the Frasnian/Famennian boundary, commonly referred to as the lower and upper Kellwasser events, many reef-building organisms, such as stromatoporoid sponges and tabulate corals, suffered severely^12^. Notably, stromatoporoid sponges became totally extinct at around the Famennian/Tournaisian boundary. This boundary corresponds to the so-called Hangenberg event marking the last spike in the Devonian extinctions. Many other benthic organisms also became extinct at this time^14^. The Hangenberg event, however, was the most severe for jawed vertebrate clades, eliminating more than 96% of species, and also leading to post-extinction global shrinkage in vertebrate size^16^.

Despite significant decline in the overall biodiversity during the Late Devonian extinctions, crinoids were one of the few invertebrate groups that were not substantially affected during this time. Noteworthy, an increase in the total number of crinoid genera, leading to the major ecological reorganization (transition from the so-called Middle Paleozoic to the Late Paleozoic Crinoid Evolutionary Fauna), occurred in the early Visean^17–20^. Indeed, recent study demonstrated that origination rates of crinoids exceeded extinction rates at around Devonian/Carboniferous boundary^18^. Notably, crinoids reached their Phanerozoic peak of generic richness and abundance in the early Mississippian, which has been referred to as the ‘Age of Crinoids’^19,20^. Yet, no studies investigated whether crinoids changed their sizes during this crucial interval. To test this we thus assembled a database comprising sizes of calyces for 262 crinoid genera occurring in the Frasnian-Visean.

## Results

**Figure 1:**
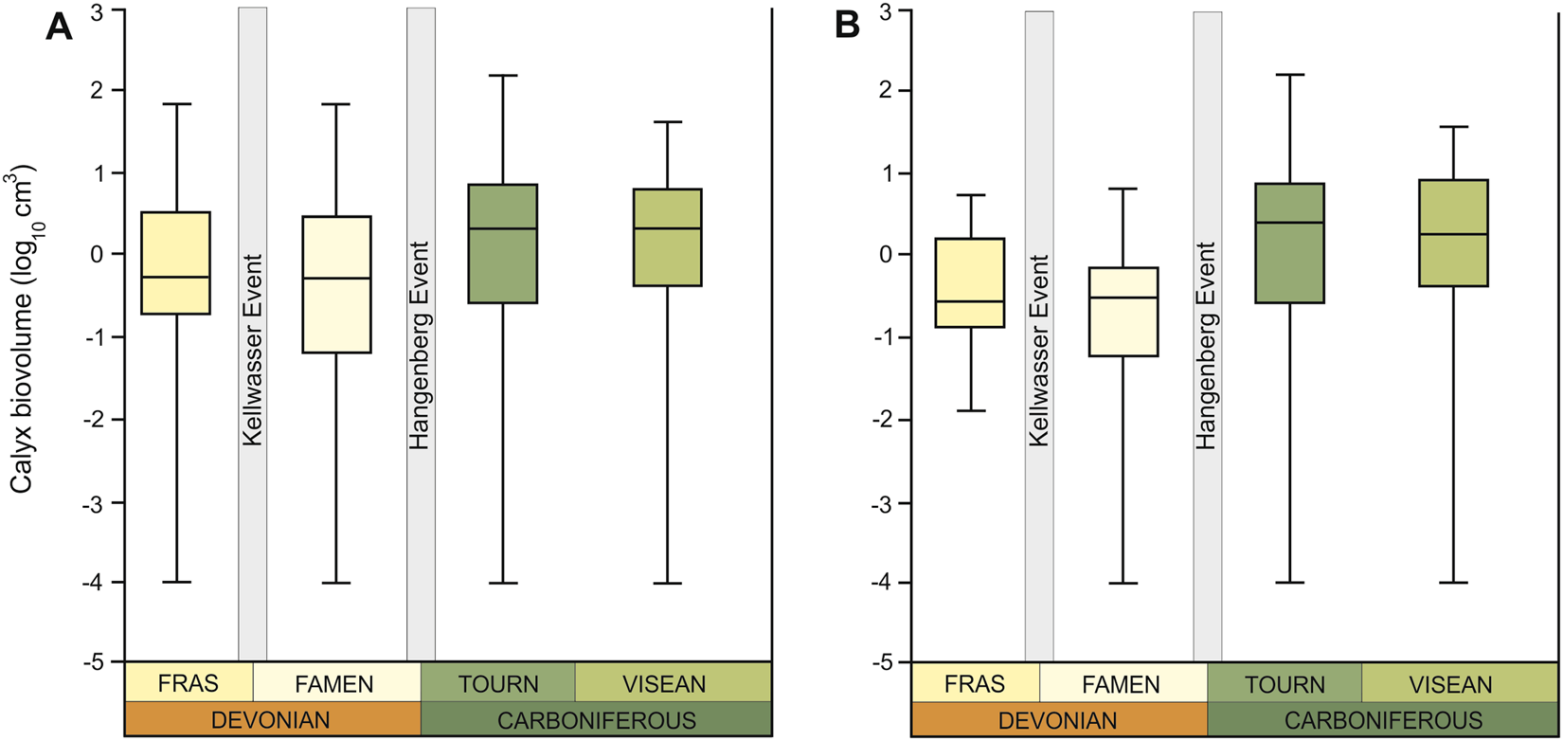
Box plots showing distribution of calyx volumes of holotypes of type species for the uppermost Devonian and lowermost Carboniferous using two different methods: “range through approach” (**A**), and “per-occurrence approach” (**B**); the 25–75 percent quartiles are drawn using a box, the median is shown with a horizontal line inside the box, the minimal and maximal values are shown with short horizontal lines (“whiskers”). Fras – Frasnian; Famen – Famennian; Tourn – Tournaisian.

**Figure 2:**
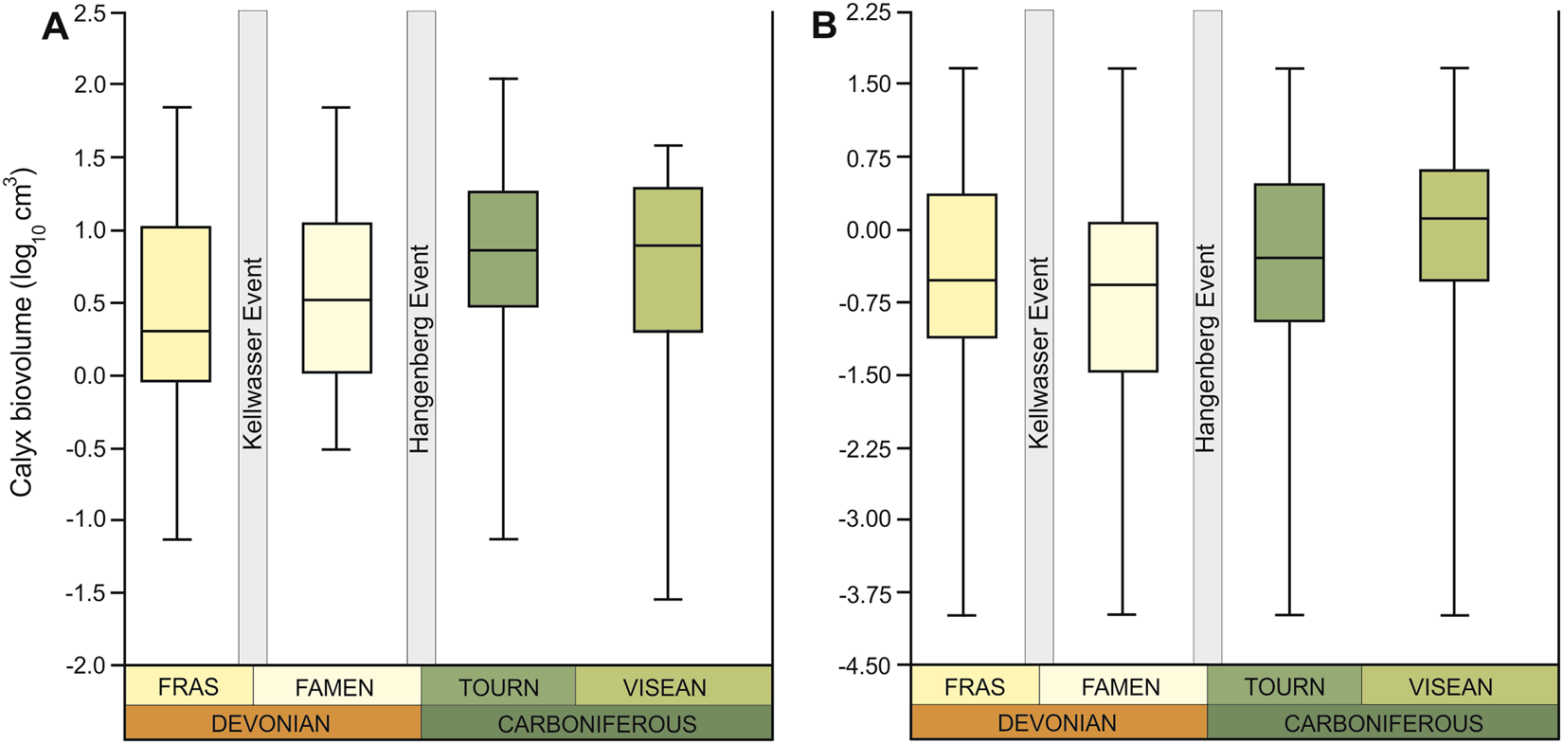
Box plots showing distribution of calyx volumes of holotypes of type species for the uppermost Devonian and lowermost Carboniferous using “range through approach” for the two sister clades Camerata (**A**) and Pentacrinoidea (**B**); the 25–75 percent quartiles are drawn using a box, the median is shown with a horizontal line inside the box, the minimal and maximal values are shown with short horizontal lines (“whiskers”). Fras – Frasnian; Famen – Famennian; Tourn – Tournaisian.

Our database shows that the median and mean size of crinoid calyces increased during the Frasnian-Visean interval (Fig. 1; Table 1). Notwithstanding the method used (details in Supplementary Materials), Frasnian and Famennian medians of log-transformed biovolumes are statistically indistinguishable from each other [Mann-Whitney *U* test; Frasnian versus Famennian: *P* = 1 (range through approach) or *P* = 1 (per-occurrence approach); details in Supplementary Tables S1–S4, S17]. By contrast, means and medians of Tournaisian and Visean sizes are much higher (Table 1; details in Supplementary Tables S2, S4, S17). The magnitude of size increase between Devonian and Carboniferous stages (Visean, in particular) is statistically significant (Table 1). The crinoid class-level trend of increasing size throughout the investigated interval is supported by linear regressions [ordinary least squares (OLS) and reduced major axis (RMA) *P* < 0.05; details in Supplementary Figs S12–S15; Tables S18–S21]. The resulting class-level size distributions (Fig. 1) using both approaches are similar and clearly show temporal heterogeneity in the variance, with an increase in the variance between the Famennian and Tournaisian. Interestingly, once the lower limit of size is reached (Frasnian or Famennian, depending on the method used), it remained constant over the study interval. Similar trends can be observed at the subclass-level, with two major sister clades (Camerata and Pentacrinoidea) displaying higher median and mean body sizes in the Carboniferous (Visean, in particular) (Table 1, Fig. 2; Supplementary Tables S5–S8, S17). However, the differences between median sizes in stages are only statistically significant for the most diverse clade – Pentacrinoidea (Fig. 2B; Table 1; Supplementary Tables S7, S8, S17). Likewise, a trend of increasing size throughout the study interval is statistically significant for Pentacrinoidea only [ordinary least squares (OLS) and reduced major axis (RMA) *P* < 0.05; see Supplementary Figs S16–S19; Tables S22–S25]. It should be noted, however, that although trends of increasing size of Camerata throughout Frasnian-Visean interval lack statistical significance (presumably due to lower number of data points), *r* values remain positive (Supplementary Figs S16–S17, Tables S22, S23). In contrast to Pentacrinoidea, for which minimum and maximum biovolumes remained stable over the study interval, the variance of Camerata reveals strong temporal heterogeneity (Fig. 2A).

**Table 1:** Descriptive statistics for crinoid biovolumes.

	Frasnian	Famennian	Tournaisian	Visean
**All crinoids [range through approach]**				
Mean biovolume [cm^3^]	4.6484	4.3048	7.2715	5.7685
Median biovolume [cm^3^]	0.5539	0.5539	1.9212	1.9177
log_10_	−0.2566^a^	−0.2566^a^	0.2828^a,b^	0.2824^b^
SE	0.1399	0.1514	0.0970	0.0850
N	59	67	142	162
**All crinoids [per occurrence approach]**				
Mean biovolume [cm^3^]	1.06527	0.8478308	7.436411	5.9493
Median biovolume [cm^3^]	0.2508	0.2740	2.5837	1.6909
log_10_	−0.6007^a^	−0.5622^a^	0.4122^b^	0.2279^b^
SE	0.1629	0.3283	0.1079	0.1097
N	21	13	111	92
**Camerata [range through approach]**				
Mean biovolume [cm^3^]	9.463642	10.43709	14.04262	11.36096
Median biovolume [cm^3^]	2.2533	3.2120	6.8823	6.7030
log_10_	0.3528^a^	0.5067^a^	0.8377^a^	0.8261^a^
N	19	18	57	44
**Pentacrinoidea [range through approach]**				
Mean biovolume [cm^3^]	2.3612	2.0522	2.7308	3.6831
Median biovolume [cm^3^]	0.3249	0.2814	0.4754	1.2874
log_10_	−0.4891^a^	−0.5507^a^	−0.3229^a,b^	0.1097^b^
N	40	49	85	118
**Disparida [range through approach]**				
Mean biovolume [cm^3^]	0.3922	0.3079	0.4721	0.7576
Median biovolume [cm^3^]	0.3589	0.3449	0.0356	0.0292
log_10_	−0.4454^a^	−0.4623^a^	−1.5494^a^	−1.5496^a^
N	6	9	8	10
**Cladida [range through approach]**				
Mean biovolume [cm^3^]	2.7086	2.4446	2.9655	3.9906
Median biovolume [cm^3^]	0.2630	0.2777	0.4891	1.3767
log_10_	−0.5856^a^	−0.5565^a^	−0.3106^a^	0.1388^b^
N	34	40	77	107
**Eucladida [range through approach]**				
Mean biovolume [cm^3^]	0.9429	0.9076	1.9153	2.8913
Median biovolume [cm^3^]	0.2018	0.1916	0.3012	1.1237
log_10_	−0.6952^a^	−0.7200^a^	−0.5229^a^	0.0507^b^
N	24	32	62	91
**Flexibilia [range through approach]**				
Median biovolume [cm^3^]	2.0485	2.7466	2.5837	5.9020
Mean biovolume [cm^3^]	9.597	13.291	8.228	10.346
log_10_	0.2961^a^	0.4380^a^	0.4122^a^	0.7710^a^
N	10	8	15	17

Other metrics mapped in box plots (Fig. 1). Log medians sharing the same superscript are not significantly different (*p* > 0.05). SE – standard error; N – number of genera.

**Figure 3:**
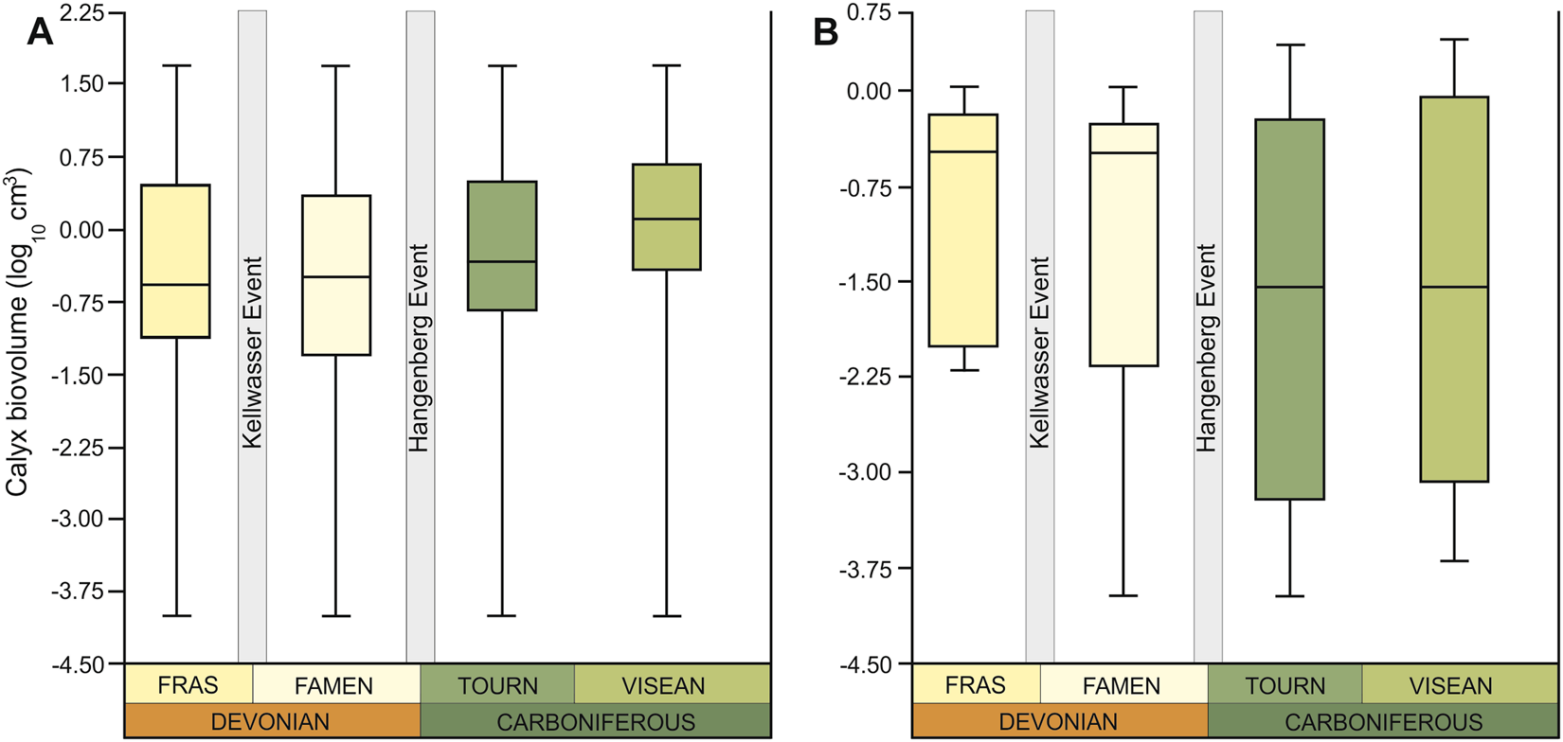
Box plots showing distribution of calyx volumes of holotypes of type species for the uppermost Devonian and lowermost Carboniferous using “range through approach” for the two sister clades Cladida (**A**) and Disparida (**B**); the 25–75 percent quartiles are drawn using a box, the median is shown with a horizontal line inside the box, the minimal and maximal values are shown with short horizontal lines (“whiskers”). Fras – Frasnian; Famen – Famennian; Tourn – Tournaisian.

**Figure 4:**
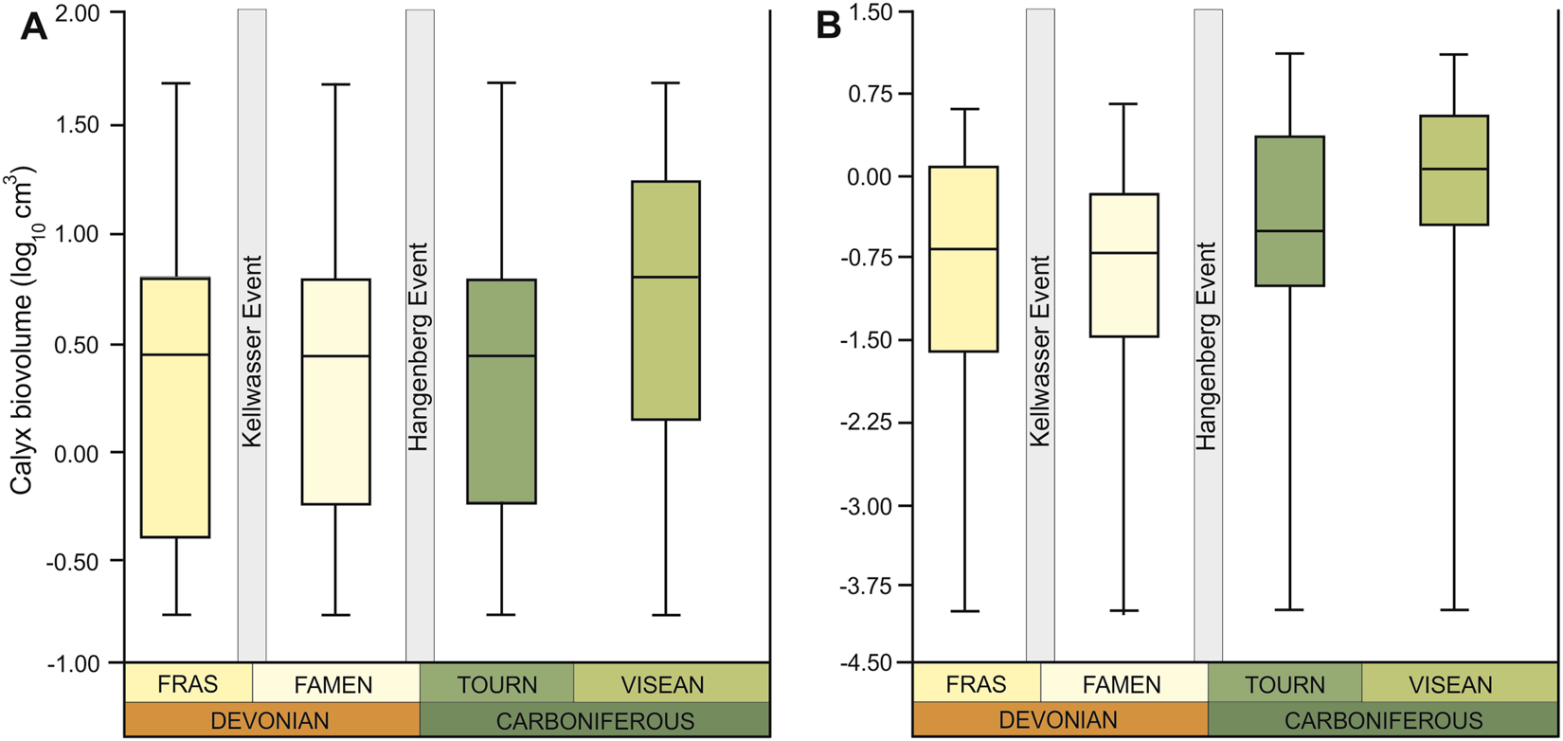
Box plots showing distribution of calyx volumes of holotypes of type species for the uppermost Devonian and lowermost Carboniferous using “range through approach” for the two sister clades Flexibilia (**A**) and Eucladida (**B**); the 25–75 percent quartiles are drawn using a box, the median is shown with a horizontal line inside the box, the minimal and maximal values are shown with short horizontal lines (“whiskers”). Fras – Frasnian; Famen – Famennian; Tourn – Tournaisian.

At the parvclass level (Cladida vs. Disparida) there are some notable differences in the body-size trends (Fig. 3; Supplementary Figs S20–S23; Tables S9–S12, S17, S26–29). Although size distributions of cladids are similar to those observed at higher taxonomic levels (Fig. 3A), disparids show lower median body sizes in the Carboniferous than in the Devonian stages (Fig. 3B) (note, however, that their mean sizes actually increase, see Table 1). Interestingly, their maximum and minimum biovolumes increased over the study interval. However, body-size trends of disparids, which are a low-diversity goup (only represented by several genera in the study interval), should be treated with caution. Given such scanty data, firm statistical conclusions cannot be obtained (Table 1, Supplementary Table 17). At lower taxonomic level (superorder-magnorders: Flexibilia vs. Eucladida), the patterns of size distribution are very similar to each other (Supplementary Figs S24–S27; Tables S13–S17, S30–S33), and are comparable to those seen at higher taxonomic levels (Fig. 4).

## Discussion

It has been argued that large organisms are more vulnerable to environmental stress and extinction^6^. Not surprisingly, size reduction occurred in the aftermath of major Phanerozoic extinctions^8^, and has been documented in a variety of groups, including echinoderms^21–24^. In the aftermath of the end-Devonian extinction, it has been recently determined that vertebrates experienced long-term reduction in body size^16^. The appearance of post-extinction size reduction in crinoids during this crucial time was thus expected. However, the observed trends of increasing mean crinoid body size do not match these predictions. This is surprising because it has been argued that such trends are expected to occur during stable times at some distance from recovery intervals^16^. Despite hypoxic/anoxic events, global carbonate crisis and perturbation of the global carbon cycles associated with the Late Devonian extinction events^14^, crinoids not only were diversifying markedly, experiencing only background extinction^19,20^, but also exhibited a trend toward larger mean sizes at the macroevolutionary scale. Notwithstanding, some clade-dependent (Fig. 3B) and/or short-term within-lineage size decrease (not visible at the scale of this study) associated with these extinctions cannot be excluded.

The observed class-level pattern is not consistent with the existence of an active, driven trend. Instead, a pattern, where both the mean and variance increase over evolutionary time without changing minimum size, suggests a passive Brownian diffusion-like process away from a lower size bound^25^. Interestingly, recent study demonstrated that the morphologic diversification within the eucladid subclade during the Devonian-early Carboniferous can be also characterized by the Brownian diffusion-like trajectory^26^. At the lower taxonomic level, the body size distributions either resemble Brownian diffusion model or random walks and stasis. However, due to the small number of bins, individual statistical model-fitting approaches^25^, enabling detection of directional trends in time series, cannot be performed.

It has been hypothesized that the early Mississippian radiation of crinoids resulted from multiple factors^20^: (i) expansion of Tournaisian carbonate-ramp settings following the end-Frasnian extinction of coral-stromatoporoid reefs; (ii) predatory release in the Tournaisian after the end-Famennian extinction of durophagous fishes, and (iii) increased primary productivity in the Tournaisian. To some extent the same factors might have also contributed to the passive expansion of crinoids into larger body-size niches. Additionally, increased mean size in some crinoids, although likely not actively driven, might have been also beneficial against the newly evolving Mississippian-style fish predators. Following the Hangenberg large-scale extinction of shearing fish predators, a number of unique and novel fish taxa with crushing dentition diversified in the Mississippian inducing escalatory evolution among benthic invertebrates^17,27,28^. Indeed, many innovations that potentially reflected anti-predatory adaptations were recognized. Among them are: (i) semi-infaunal lifestyle and increases in ornamentation and spinosity in brachiopods^29^, (ii) shell reinforcement and increase in shell size in bivalves^30^, (iii) origins of infaunal life habit in gastropods^30^. Anti-crushing defences in the calyx have been also documented in the Mississippian camerates^31,32^. Interestingly, some authors^33^ argued that increased predation pressure from the Mississippian-style durophagous fishes also led to a size refuge by increasing effective theca size of two early Mississipian crinoid genera (*Agaricocrinus*, *Dorycrinus*).

## Methods

We compiled a database of calyx sizes for 262 crinoid genera occurring in the Frasnian-Visean interval (details in Supplementary Materials). Calyx, defined from the top of the stalk to the position where the arms become free, is the most important morphological element in crinoids. It contains most of the visceral organs and tissues. Crinoid calyces commonly display high fossilization potential and are of diagnostic importance. Not surprisingly, the crinoid calyx is considered a good proxy for the overall crinoid body size^21^. Biovolume of calyces were estimated from published figures of type species of holotypes using standard volume calculations for different geometric solids (Supplementary Figs S1–S11). The type species of holotypes is widely considered an unbiased estimate of the median body size of species within a genus^34^. Furthermore, the inclusion of image-derived data in macroevolutionary studies is considered biologically meaningful^34^, even though such an approach is affected by a number of biases, which are, however, small and consistent across time and taxa. Two approaches were used in our analyses. In the first approach, we used only one volume estimate for the entire stratigraphic range of a given genus following proposed methodology^4^. This approach assumes that the size of the holotype of type species is representative for the genus throughout its duration. We also applied a per-occurrence and per-genus approach in that we compared body sizes of the holotypes of type species described from the Frasnian-Visean interval only (237 specimens in total), and treated all body size estimations as independent data points (i.e., without artificial extension of the crinoid biovolume of the type species throughout the entire stratigraphic range of genus). All estimated calyx volumes were subjected to various statistical tests (Shapiro-Wilk normality test, Mann-Whitney U-tests for pairwise stages with Bonferroni correction, significance levels α = 0.05) and linear regressions [Ordinary Least Squares (OLS) and Reduced Major Axis (RMA)]. Comparisons were also made between sister clades^35^, which are nested at different taxonomic levels to further dissect which (if any) lineage(s) are driving the overall pattern and/or if any lineages are characterized by dynamics that differ from the predominant trend among the Crinoidea. For a more detailed methodology see Supplementary Materials.

## Electronic supplementary material

Supplementary materials
