# Supplementary material for: Body-size increase in crinoids following the end-Devonian mass extinction
Source: Sci Rep. 2018 Jun 25;8:9606. doi: 10.1038/s41598-018-27986-x (PMC6018515; doi:10.1038/s41598-018-27986-x)
Supplement: Supplementary file 1 — Supplementary materials [file 41598_2018_27986_MOESM1_ESM.pdf]

Body-size increase in crinoids across the end-Devonian mass extinction

Krzysztof R. Brom[1,2], Mariusz A. Salamon[1,2] and Przemysław Gorzelak[3]*

Supplementary Information

Materials and methods

Calyx volume estimation

Measurements of calyces of holotypes of type species were taken in order to estimate their volumes. The measurements were made by using a caliper with a measuring error of 0.01 mm. The general volume of the calyx was approximated with standard volume calculations for different geometric solid following published methodology[1]. The calyx shape was classified into the following geometric figures: sphere (Supplementary Fig 1), hemisphere (Supplementary Fig 2), elongated spheroid (prolate) (Supplementary Fig 3), half of elongated spheroid (Supplementary Fig 4), flattened spheroid (oblate) (Supplementary Fig 5), half of flattened spheroid (Supplementary Fig 6), cone (Supplementary Fig 7), truncated cone (Supplementary Fig 8), cylinder (Supplementary Fig 9) and combine spherical solid figures (Supplementary Fig 10, 11). In addition, the calyxes of some type species display a slightly more complex shapes, such as a combination of the abovementioned solid figures, e.g., a truncated cone with a hemisphere or two truncated cones connected by a larger base. The following mathematical formulas were used to estimate the volume of calyxes displaying different shapes:

- sphere – $V = \frac{4}{3}\pi r^3$;
- hemisphere – $V = \frac{2}{3}\pi r^3$;
- elongated spheroid (prolate) – $V = \frac{4}{3}\pi a^2 c$;
- half of elongated spheroid – $V = \frac{2}{3}\pi a^2 c$;
- flattened spheroid (oblate) – $V = \frac{4}{3}\pi a^2 c$;
- half of flattened spheroid – $V = \frac{2}{3}\pi a^2 c$;
- cone – $V = \frac{1}{3}\pi r^2 h$;
- truncated cone – $V = \frac{1}{3}\pi h(R^2 + Rr + r^2)$;
- cylinder – $V = \pi r^2 h$.

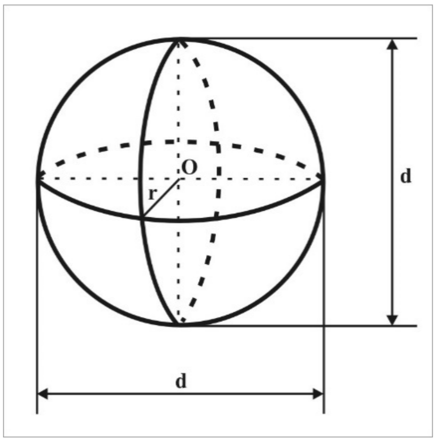

Supplementary Fig 1. Schematic drawing of sphere with marked sections, which are necessary to estimate the volume (r – radius, d – diameter).

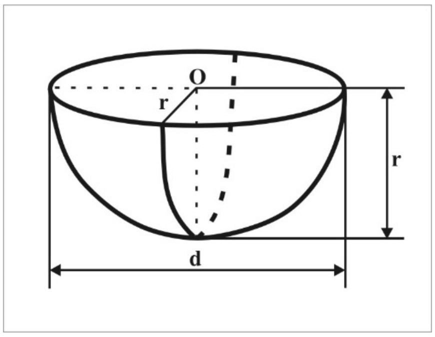

Supplementary Fig 2. Schematic drawing of hemisphere with marked sections, which are necessary to estimate the volume (r – radius, d – diameter).

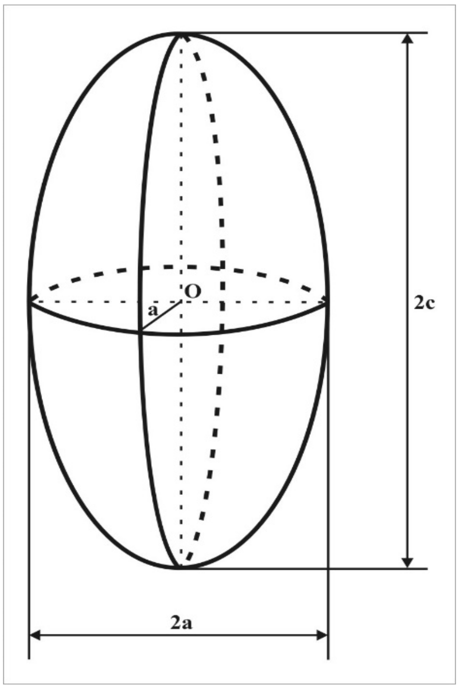

Supplementary Fig 3. Schematic drawing of elongated spheroid (prolate) with marked sections, which are necessary to estimate the volume (a – equatorial radius of the spheroid, c – distance from centre to pole along the symmetry axis).

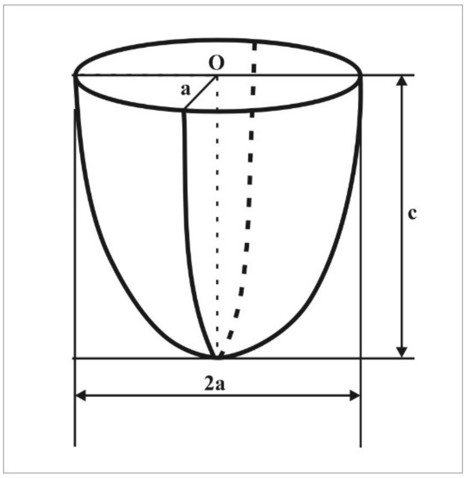

Supplementary Fig 4. Schematic drawing of half of elongated spheroid with marked sections, which are necessary to estimate the volume (a – equatorial radius of the spheroid, c – distance from centre to pole along the symmetry axis).

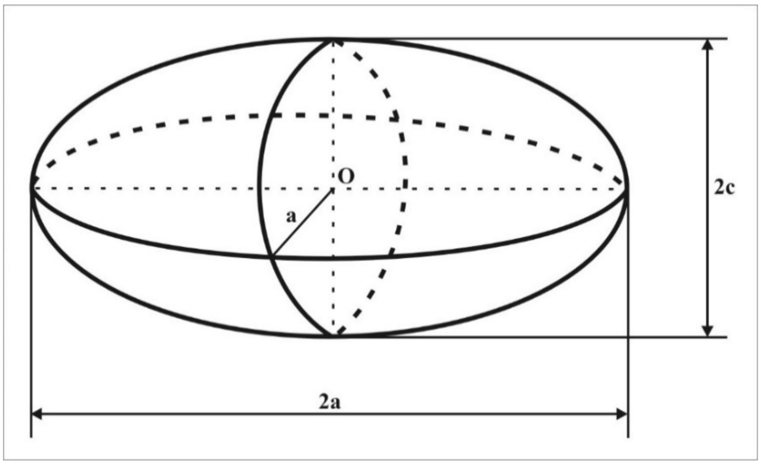

Supplementary Fig 5. Schematic drawing of flattened spheroid (oblate) with marked sections, which are necessary to estimate the volume (a – equatorial radius of the spheroid, c – distance from centre to pole along the symmetry axis).

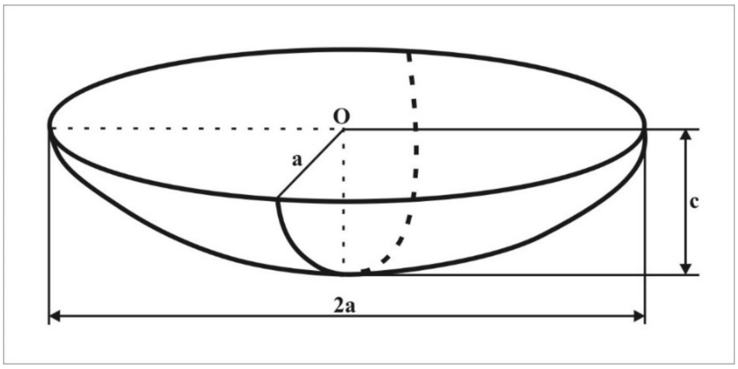

Supplementary Fig 6. Schematic drawing of half of flattened spheroid with marked sections, which are necessary to estimate the volume (a – equatorial radius of the spheroid, c – distance from centre to pole along the symmetry axis).

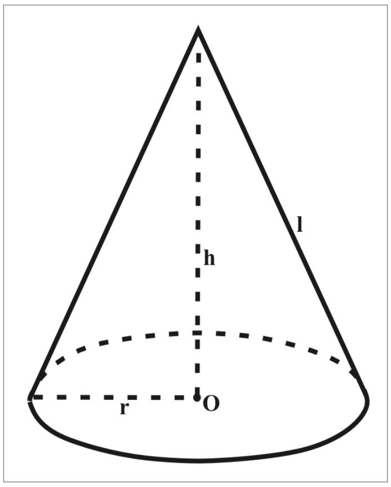

Supplementary Fig 7. Schematic drawing of cone with marked sections, which are necessary to estimate the volume (r – radius, h – height, l – slant height).

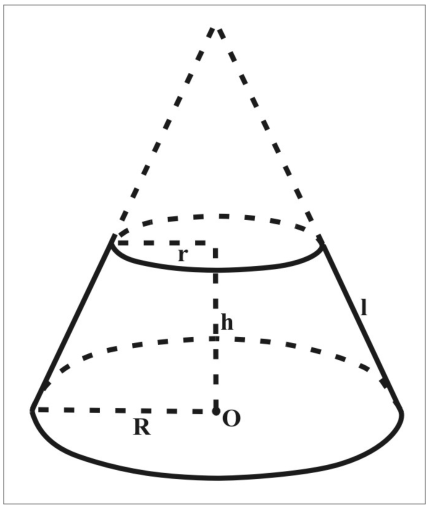

Supplementary Fig 8. Schematic drawing of truncated cone with marked sections, which are necessary to estimate the volume (r – smaller radius, R – bigger radius, h – height, l – slant height).

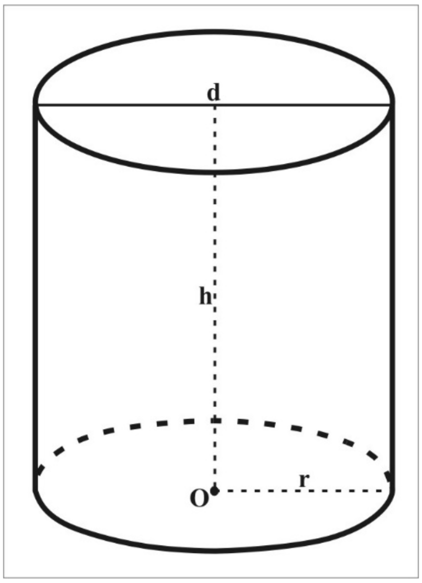

Supplementary Fig 9. Schematic drawing of cylinder with marked sections, which are necessary to estimate the volume (r – radius, d – diameter, h – height).

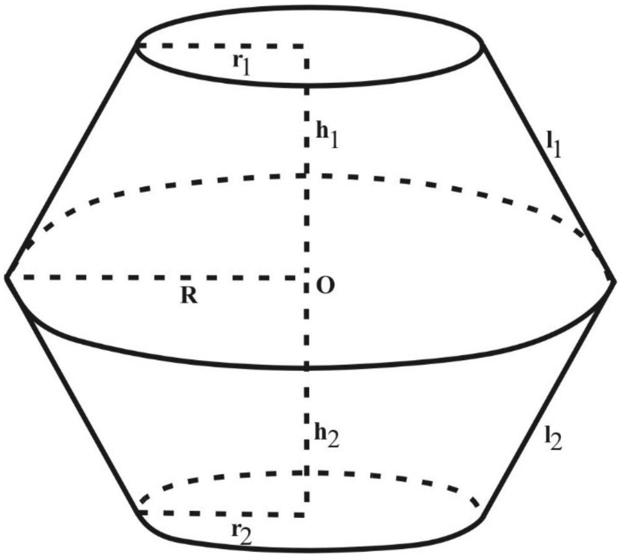

Supplementary Fig 10. Schematic drawing of combine spherical solid figures (two truncated cones connected by a larger base) with marked sections, which are necessary to estimate the volume (r_1, r_2 – smaller radii, R – bigger radius, h_1, h_2 – heights, l – slant heights).

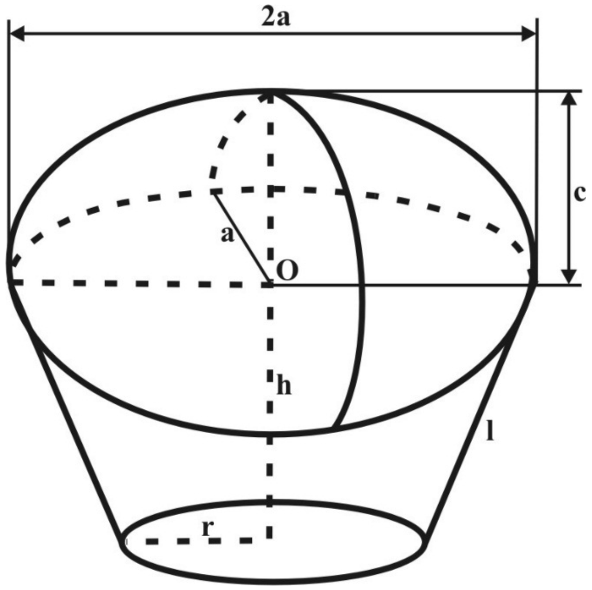

Supplementary Fig 11. Schematic drawing of combine spherical solid figures truncated cone with a half of flattened spheroid) with marked sections, which are necessary to estimate the volume (r – smaller radius, R – bigger radius, h – height, l – slant height, a – equatorial radius of the spheroid, c – distance from centre to pole along the symmetry axis).

The measurements of calyces were mostly made from the Treatise on Invertebrate Paleontology[2]. However, when illustrations from the Treatise were insufficient to estimate the volume of calyx, we also used primary literature[3-23]. Measurements of recently described taxa were also taken from published source papers. Our dataset contains body sizes for 262

Frasnian-Visean crinoid genera (Supplementary Table 34). This dataset is estimated to contain 93,24% of all described crinoid genera from this interval (cf., the newest crinoid database[24]). Remaining named genera lack sufficient descriptions and/or figures.

Two different approaches were applied to analyze crinoid body trends. In the first approach, we used only one volume estimate for the entire stratigraphic range of a given genus[25]. This approach assumes that the size of the holotype of the type species is representative for the genus throughout its duration. Such an approach has been commonly used in paleontological literature and seems also valid at the scale of this study given that size variation among genera is greater than size variation within genera[25-27]. However, given that some crinoid genera may potentially display temporal size changes[28,29], we also applied a per-occurrence and per-genus approach in that we compared body sizes of the holotypes of the type species recorded from the Frasnian-Visean interval only (237 specimens), and treated all body size estimations as independent data points (i.e., without artificial extension of the calyx volume estimation of the type species throughout the entire stratigraphic range of the genus).

As noted above, crinoid body sizes were estimated from published figures of the holotypes of type species, which is considered an unbiased estimate of the median body size of species within a genus[30]. Although size measurements of figured specimens in monographs can be biased by a number of factors, the bias is very small and consistent across time and taxa, thus the inclusion of image-derived data in macroevolutionary studies is considered biologically meaningful[31]. Indeed our image-derived calyx volumes are essentially identical to those estimated from the real specimens of type species deposited in the Natural History Museum in Berlin (Germany) and the Natural History Museum in London (UK). However, the volumes obtained from the measurements of real specimens were not taken into account for statistical analysis because application of such a “mixed” approach in the same analysis may introduce bias[30].

Statistical analysis

All estimated calyx volumes were subjected to various statistical tests in the PAST software (PAleontological STatistics 3.18 version)[32]. The data were first transformed by Log_{10} to reduce the effects of allometry.

The first dataset comprising 262 volumes (with artificial extension of calyx volume for the entire stratigraphic range of a given genus) was subjected to normality tests (Shapiro-Wilk; significance level $\alpha = 0.05$) in order to determine whether a parametric or nonparametric test is appropriate. All four groups of volumes binned by stage did not exhibit

normal distribution, therefore non-parametric tests were used for further analyses (Supplementary Table 1). Results of Mann-Whitney U-tests for pairwise stages with Bonferroni correction (significance level $\alpha = 0.05$) are presented in Supplementary Table 2.

Supplementary Table 1. Results of Shapiro-Wilk tests for calyx volumes of holotypes of type species binned to 4 stages in which given genera occurred (using a "range through approach"); N – number of data, *p*-value – probability value, * – indicates statistically significant result (*p*-value<0.05).

	**N**	***p*-value**
**Frasnian**	59	4.633E-14*
**Famennian**	67	3.91E-15*
**Tournaisian**	142	4.689E-21*
**Visean**	162	4.491E-17*

Supplementary Table 2. *p*-values obtained from U Mann-Whitney test for calyx volumes of holotypes of type species binned to 4 stages in which given genera occurred (using a "range through approach"); * – indicates statistically significant result (*p*-value<0.05).

	**Frasnian**	**Famennian**	**Tournaisian**	**Visean**
**Frasnian**	–	1	0.2335	0.04053*
**Famennian**		–	0.07611	0.01007*
**Tournaisian**			–	1
**Visean**				–

The same tests were applied to the second dataset comprising the estimated calyx volumes of holotypes of type species described from the Frasnian-Visean interval only, without artificial extension of the crinoid volume estimation of a given genus throughout its entire stratigraphic range ("per-occurrence approach"). The results of statistical analyses are presented in Supplementary Table 3 and 4.

Supplementary Table 3. Results of Shapiro-Wilk test for calyx volumes of holotypes of type species binned to 4 stages in which given type species was described (using a “per-occurrence approach”); N – number of data, *p*-value – probability value, * – indicates statistically significant result (*p*-value<0.05).

	**N**	***p*-value**
**Frasnian**	21	6.732E-05*
**Famennian**	13	8.001E-06*
**Tournaisian**	111	3.465E-19*
**Visean**	92	4.12E-12*

Supplementary Table 4. *p*-values obtained from U Mann-Whitney test for calyx volumes of holotypes of type species binned to 4 stages in which given type species was described (using a “per-occurrence approach”); * – indicates statistically significant result (*p*-value<0.05).

	**Frasnian**	**Famennian**	**Tournaisian**	**Visean**
**Frasnian**	–	1	0.03715*	0.0251*
**Famennian**		–	0.017*	0.0111*
**Tournaisian**			–	1
**Visean**				–

The same statistical procedures were repeated also at the lower taxonomic levels (i.e. between camerates (subclass Camerata = orders Diplobathrida and Monobathrida; Supplementary Table 5, 6) and pentacrinoids (subclass Pentacrinoidea = parvclass Disparida and parvclass Cladida; Supplementary Table 7, 8); between disparids (parvclass Disparida; Supplementary Table 9, 10) and cladides (parvclass Cladida = primitive and advanced cladids, Taxocrinida and Sagenocrinida; Supplementary Table 11, 12); and between eucladids (magnorder Eucladida = primitive and advanced cladids; Supplementary Table 13, 14) and flexibiles (superorder Flexibilia = order Taxocrinida and Sagenocrinida; Supplementary Table 15, 16)). In these analyses we only used a database comprising volumes of the holotypes of type species extended throughout the entire stratigraphic range of a given genus because it contains sufficient number of datapoints allowing statistical comparisons at a lower taxonomic level. Supplementary Table 17 summarizes all results obtained from U Mann-Whitney tests.

Supplementary Table 5. Shapiro-Wilk test results for calyx volumes of holotypes of type species of Camerata binned to 4 stages in which given genera occurred (using a "range through approach"); N – number of data, *p*-value – probability value, * – indicates statistically significant result (*p*-value<0.05).

	**N**	***p*-value**
**Frasnian**	19	1.622E-06*
**Famennian**	18	3.601E-06*
**Tournaisian**	57	2.818E-12*
**Visean**	44	0.0001645*

Supplementary Table 6. *p*-values obtained from U Mann-Whitney test adopted for calyx volumes of holotypes of type species of Camerata binned to 4 stages in which given genera occurred (using a "range through approach"); * – indicates statistically significant result (*p*-value<0.05).

	**Frasnian**	**Famennian**	**Tournaisian**	**Visean**
**Frasnian**	–	1	0.353	0.606
**Famennian**		–	1	1
**Tournaisian**			–	1
**Visean**				–

Supplementary Table 7. Shapiro-Wilk test results for calyx volumes of holotypes of type species of Pentacrinoidea binned to 4 stages in which given genera occurred (using a "range through approach"); N – number of data, *p*-value – probability value, * – indicates statistically significant result (*p*-value<0.05).

	**N**	***p*-value**
**Frasnian**	40	1.119E-12*
**Famennian**	49	3.504E-14*
**Tournaisian**	85	1.495E-16*
**Visean**	118	7.321E-17*

Supplementary Table 8. *p*-values obtained from U Mann-Whitney test adopted for calyx volumes of holotypes of type species of Pentacrinoidea binned to 4 stages in which given genera occurred (using a “range through approach”); * – indicates statistically significant result (*p*-value<0.05).

	**Frasnian**	**Famennian**	**Tournaisian**	**Visean**
**Frasnian**	–	1	1	0,02378*
**Famennian**		–	1	0.001889 *
**Tournaisian**			–	0,05014*
**Visean**				–

Supplementary Table 9. Shapiro-Wilk test results for calyx volumes of holotypes of type species of Disparida binned to 4 stages in which given genera occurred (using a “range through approach”); N – number of data, *p*-value – probability value, * – indicates statistically significant result (*p*-value<0.05).

	**N**	***p*-value**
**Frasnian**	6	2.945E-01*
**Famennian**	9	3.409E-02*
**Tournaisian**	8	1.149E-04*
**Visean**	10	1.384E-04*

Supplementary Table 10. *p*-values obtained from U Mann-Whitney test adopted for calyx volumes of holotypes of type species of Disparida binned to 4 stages in which given genera occurred (using a “range through approach”); * – indicates statistically significant result (*p*-value<0.05).

	**Frasnian**	**Famennian**	**Tournaisian**	**Visean**
**Frasnian**	–	1	1	1
**Famennian**		–	1	1
**Tournaisian**			–	1
**Visean**				–

Supplementary Table 11. Shapiro-Wilk test results for calyx volumes of holotypes of type species of Cladida binned to 4 stages in which given genera occurred (using a “range through approach”); N – number of data, *p*-value – probability value, * – indicates statistically significant result (*p*-value<0.05).

	**N**	***p*-value**
**Frasnian**	34	1.770E-11
**Famennian**	40	1.454E-12
**Tournaisian**	77	1.571E-15
**Visean**	107	9.605E-16

Supplementary Table 12. *p*-values obtained from U Mann-Whitney test adopted for calyx volumes of holotypes of type species of Cladida binned to 4 stages in which given genera occurred (using a “range through approach”); * – indicates statistically significant result (*p*-value<0.05).

	**Frasnian**	**Famennian**	**Tournaisian**	**Visean**
**Frasnian**	–	1	1	0.01909*
**Famennian**		–	1	0.00329*
**Tournaisian**			–	0.02932*
**Visean**				–

Supplementary Table 13. Shapiro-Wilk test results for calyx volumes of holotypes of type species of Eucladida binned to 4 stages in which given genera occurred (using a “range through approach”); N – number of data, *p*-value – probability value, * – indicates statistically significant result (*p*-value<0.05).

	**N**	***p*-value**
**Frasnian**	24	1,580E-06*
**Famennian**	32	3,321E-08*
**Tournaisian**	62	1,316E-12*
**Visean**	91	1,476E-12*

Supplementary Table 14. *p*-values obtained from U Mann-Whitney test adopted for calyx volumes of holotypes of type species of Eucladida binned to 4 stages in which given genera occurred (using a "range through approach"); * – indicates statistically significant result (*p*-value<0.05).

	**Frasnian**	**Famennian**	**Tournaisian**	**Visean**
**Frasnian**	–	1	1	0,006015*
**Famennian**		–	0,7768	0,0008316*
**Tournaisian**			–	0,02925*
**Visean**				–

Supplementary Table 15. Shapiro-Wilk test results for calyx volumes of holotypes of type species of Flexibilia binned to 4 stages in which given genera occurred (using a "range through approach"); N – number of data, *p*-value – probability value, * – indicates statistically significant result (*p*-value<0.05).

	**N**	***p*-value**
**Frasnian**	10	2.744E-06*
**Famennian**	8	3.076E-05*
**Tournaisian**	15	1.719E-05*
**Visean**	17	0.000237*

Supplementary Table 16. *p*-values obtained from U Mann-Whitney test adopted for calyx volumes of holotypes of type species of Flexibilia binned to 4 stages in which given genera occurred (using a "range through approach"); * – indicates statistically significant result (*p*-value<0.05).

	**Frasnian**	**Famennian**	**Tournaisian**	**Visean**
**Frasnian**	–	1	1	0.7526
**Famennian**		–	1	1
**Tournaisian**			–	1
**Visean**				–

Supplementary Table 17. Summary table showing *p*-values obtained from U Mann-Whitney tests; * – indicates statistically significant result (*p*-value<0.05).

	Frasnian-Famennian	Frasnian-Tournaisian	Frasnian-Visean	Famennian-Tournaisian	Famennian-Visean	Tournaisian-Visean
all crinoids ("range through approach")	1	0.2335	0.04053*	0.07611	0.01007*	1
all crinoids ("per-occurrence approach")	1	0.03715*	0.0251*	0.017*	0.0111*	1
Subclass Camerata ("range through approach")	1	0.353	0.606	1	1	1
Subclass Pentacrinoidea ("range through approach")	1	1	0.02378*	1	0.001889*	0.05014
Parvclass Disparida ("range through approach")	1	1	1	1	1	1
Parvclass Cladida ("range through approach")	1	1	0.01909*	1	0.00329*	0.02932*
Magnorder Eucladida ("range through approach")	1	1	0.006015*	0.7768	0.0008316*	0.02925*
Superorder Flexibilla ("range through approach")	1	1	1	0.7526	1	1

Linear regressions (Ordinary Least Squares (OLS) and Reduced Major Axis (RMA) were used to determine the significance and effect size of correlations between log-transformed volume of crinoid calyx and age (the midpoints of our time bins) (Supplementary Fig 12-27; Supplementary Tables 18-33). This was done for the overall dataset (using two approaches) and subsets (using the first approach).

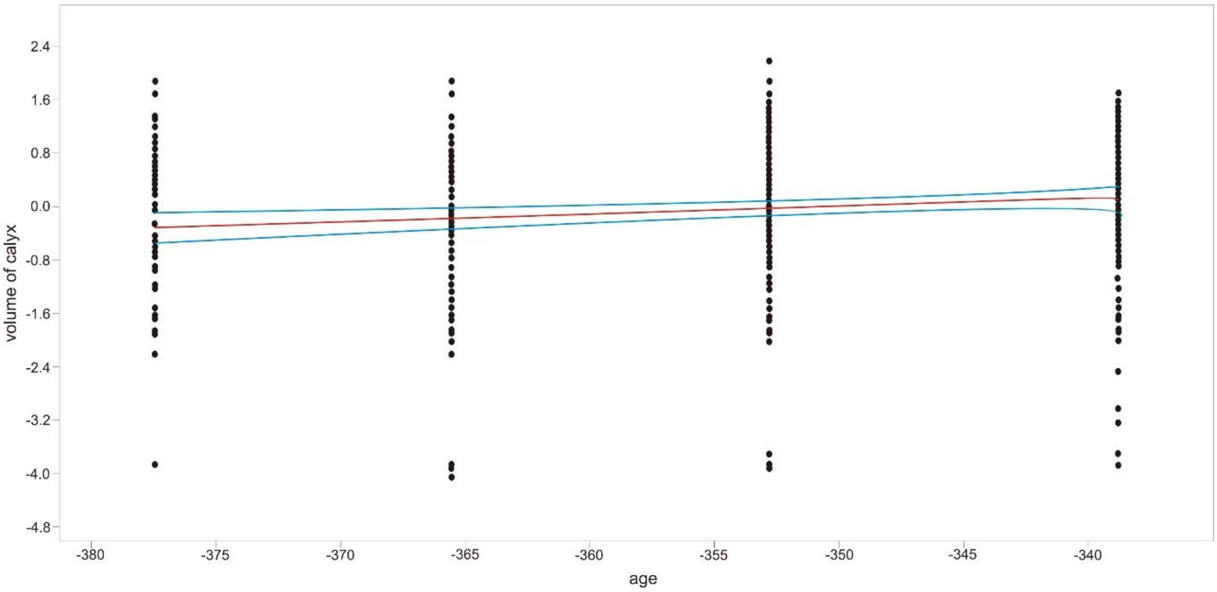

Supplementary Fig 12. OLS regression: Late Devonian-Early Carboniferous Log volume/age. [holotypes of type species of all crinoids binned to 4 stages in which given genera occurred (using a "range through approach")].

Supplementary Table 18. OLS regression statistics: Late Devonian-Early Carboniferous sizes and age. [holotypes of type species of all crinoids binned to 4 stages in which given genera occurred (using a "range through approach")]. Confidence intervals from 1999 bootstrapped replicates. Body volume transformed by Log_{10}.

**Slope *a***	0.011691	Std. error *a*	0.0040206
**Intercept *b***	4.0878	Std. error *b*	1.4199
95% bootstrapped confidence intervals (N=1999)			
**Slope *a***		(0.0038873, 0.019287)	
**Intercept *b***		(1.3485, 6.7723)	
Correlation			
r		0.13918	
***r*²**		0.019371	
t		2.9077	
***p* (uncorr.)**		0.0038307	
**permutation *p***		0.0048	

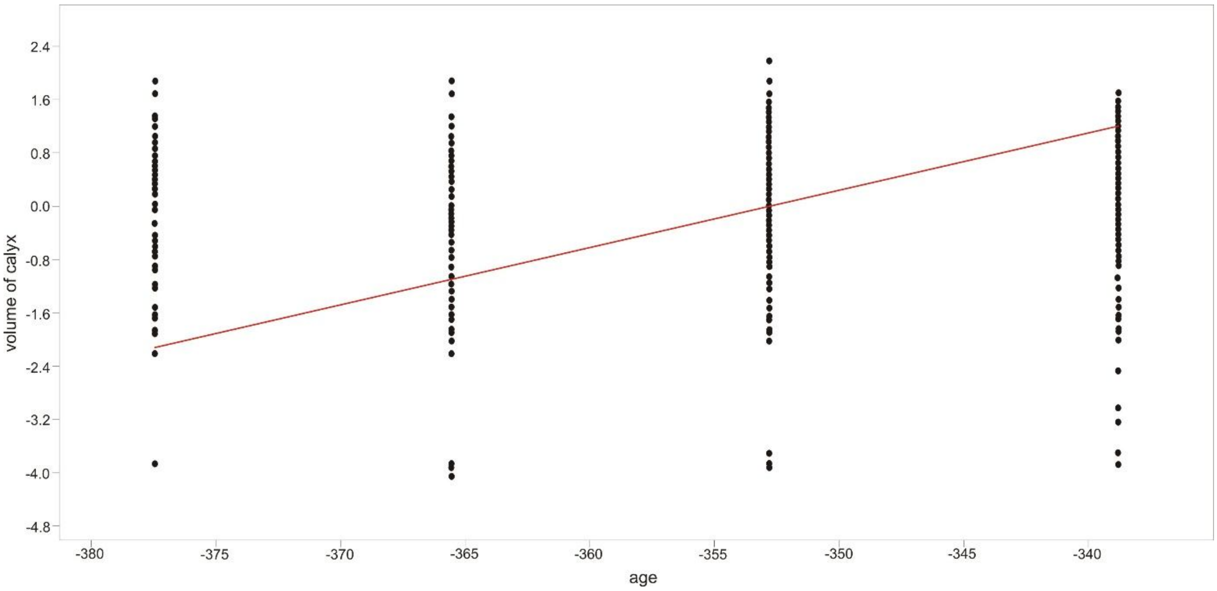

Supplementary Fig 13. RMA regression: Late Devonian-Early Carboniferous Log volume/age [holotypes of type species of all crinoids binned to 4 stages in which given genera occurred (using a "range through approach")].

Supplementary Table 19. RMA regression statistics: Late Devonian-Early Carboniferous sizes and age. [holotypes of type species of all crinoids binned to 4 stages in which given genera occurred (using a "range through approach")]. Confidence intervals from 1999 bootstrapped replicates. Body volume transformed by Log_{10}.

**Slope *a***	0.083996	Std. error *a*	0.0040206
**Intercept *b***	29.604	Std. error *b*	2.0183
95% bootstrapped confidence intervals (N=1999)			
**Slope *a***		(0.075421, 0.092924)	
**Intercept *b***		(26.645, 32.681)	
Correlation			
r		0.13918	
r r^2		0.019371	
t		2.9077	
***p* (uncorr.)**		0.0038307	
**permutation *p***		0.0039	

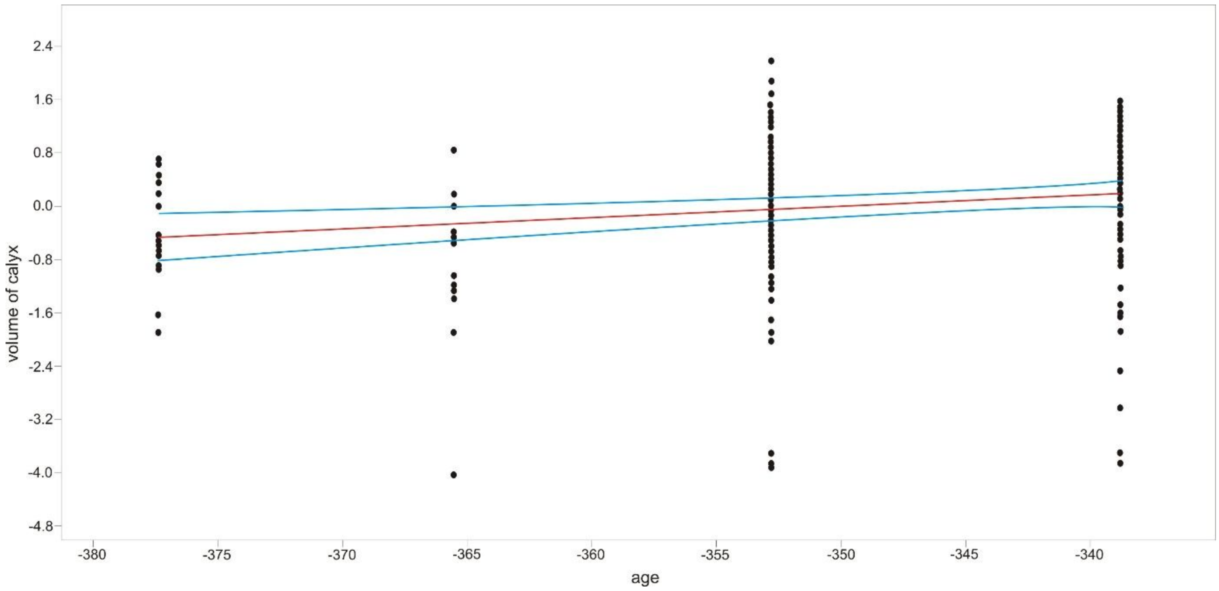

Supplementary Fig 14. OLS regression: Late Devonian-Early Carboniferous Log volume/age. [holotypes of type species of all crinoids binned to 4 stages in which given genera occurred (using a "per-occurrence approach")].

Supplementary Table 20. OLS regression statistics: Late Devonian-Early Carboniferous sizes and age. [holotypes of type species of all crinoids binned to 4 stages in which given genera occurred (using a "per-occurrence approach")]. Confidence intervals from 1999 bootstrapped replicates. Body volume transformed by Log_{10}.

**Slope *a***	0.017155	Std. error *a*	0.0061147
**Intercept *b***	5.9986	Std. error *b*	2.1425
95% bootstrapped confidence intervals (N=1999)			
**Slope *a***		(0.0063861, 0.027277)	
**Intercept *b***		(2.238, 9.5898)	
Correlation			
r		0.18002	
***r*²**		0.032407	
t		2.8055	
***p* (uncorr.)**		0.0054447	
**permutation *p***		0.0061	

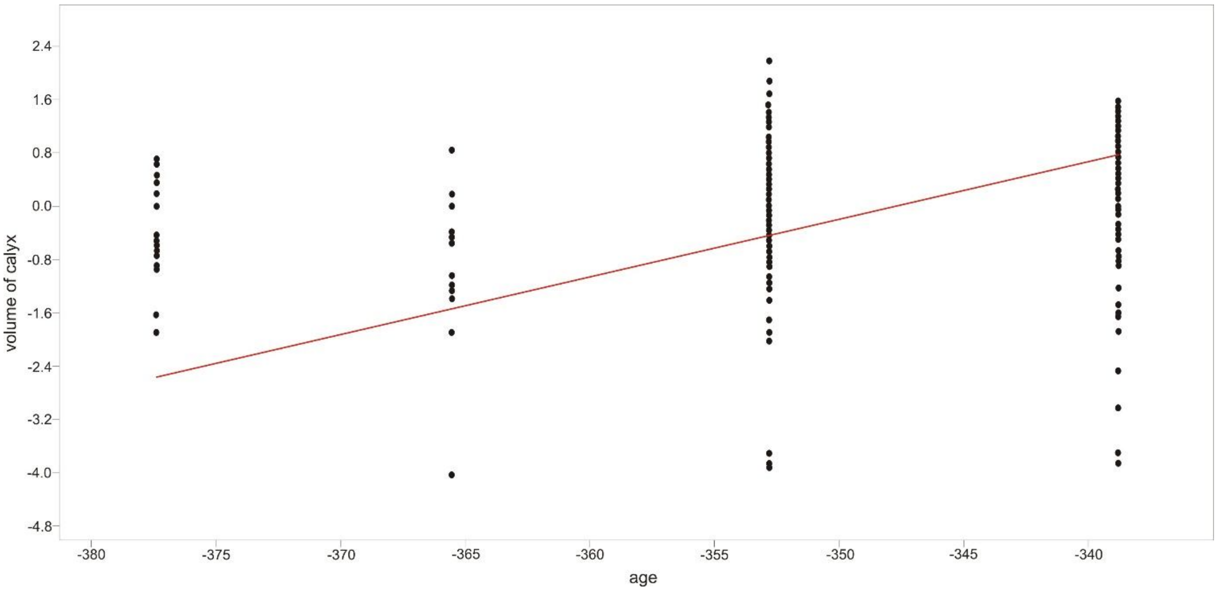

Supplementary Fig 15. RMA regression: Late Devonian-Early Carboniferous Log volume/age. [holotypes of type species of all crinoids binned to 4 stages in which given genera occurred (using a "per-occurrence approach")].

Supplementary Table 21 – RMA regression statistics: Late Devonian-Early Carboniferous sizes and age. [holotypes of type species of all crinoids binned to 4 stages in which given genera occurred (using a "per-occurrence approach")]. Confidence intervals from 1999 bootstrapped replicates. Body volume transformed by Log_{10}.

**Slope *a***	0.095294	Std. error *a*	0.0061147
**Intercept *b***	33.362	Std. error *b*	4.5936
95% bootstrapped confidence intervals (N=1999)			
**Slope *a***		(0.078699, 0.10995)	
**Intercept *b***		(27.683, 38.415)	
Correlation			
r		0.18002	
r^2		0.032407	
t		2.8055	
***p* (uncorr.)**		0.0054447	
**permutation *p***		0.0058	

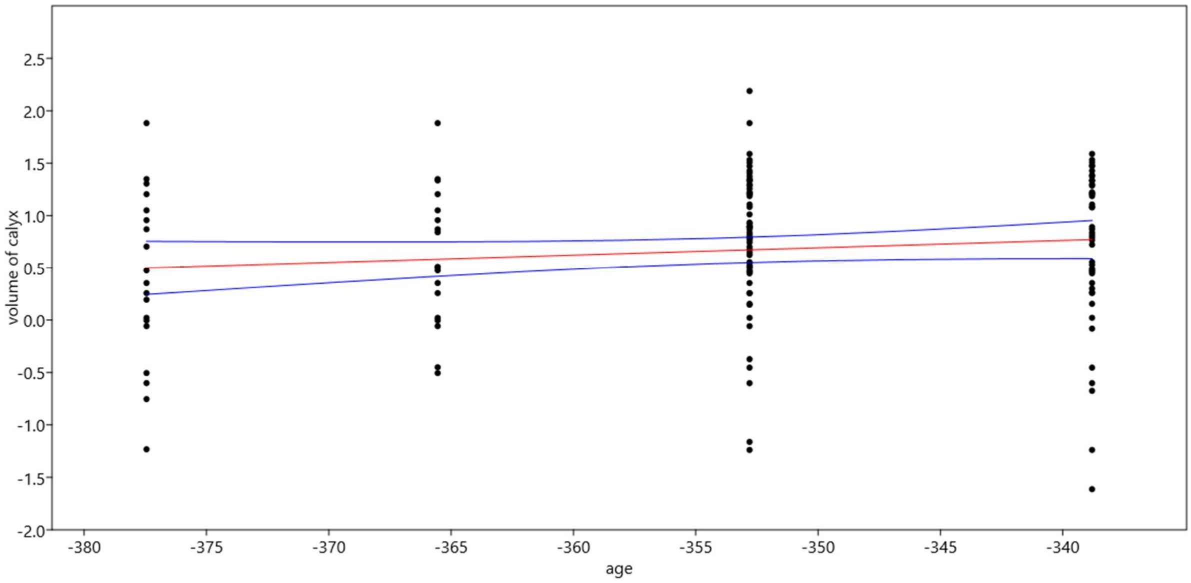

Supplementary Fig 16. OLS regression: Late Devonian-Early Carboniferous Log volume/age. [holotypes of type species of camerates binned to 4 stages in which given genera occurred using a "range through approach")].

Supplementary Table 22. OLS regression statistics: Late Devonian-Early Carboniferous sizes and age. [holotypes of type species of camerates binned to 4 stages in which given genera occurred using a "range through approach")]. Confidence intervals from 1999 bootstrapped replicates. Body volume transformed by Log_{10}.

**Slope *a***	0.0070326	Std. error *a*	0.0047207
**Intercept *b***	3.1546	Std. error *b*	1.6694
95% bootstrapped confidence intervals (N=1999)			
**Slope *a***		(-0.0030099, 0.016932)	
**Intercept *b***		(-0.37364, 6.6729)	
Correlation			
r		0.12671	
***r*²**		0.016056	
t		1.4897	
***p* (uncorr.)**		0.13861	
**permutation *p***		0.1375	

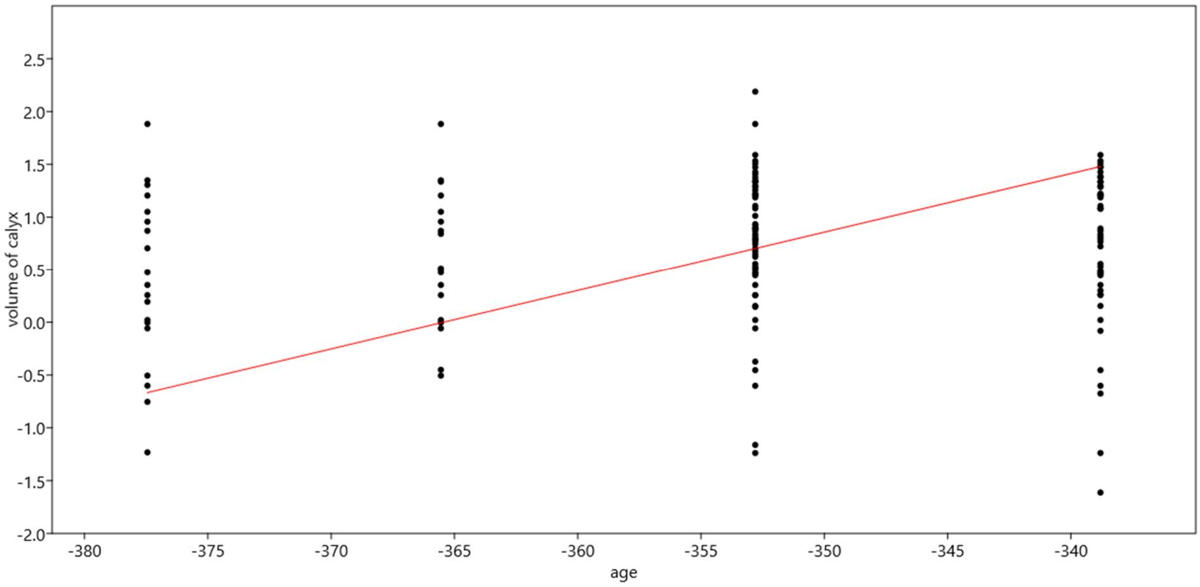

Supplementary Fig 17. RMA regression: Late Devonian-Early Carboniferous Log volume/age. [holotypes of type species of camerates binned to 4 stages in which given genera occurred using a "range through approach")].

Supplementary Table 23. RMA regression statistics: Late Devonian-Early Carboniferous sizes and age. [holotypes of type species of camerates binned to 4 stages in which given genera occurred using a "range through approach")]. Confidence intervals from 1999 bootstrapped replicates. Body volume transformed by Log_{10}.

**Slope *a***	0.0555	Std. error *a*	0.0047207
**Intercept *b***	20.283	Std. error *b*	2.7897
95% bootstrapped confidence intervals (N=1999)			
**Slope *a***		(0.046865, 0.17152)	
**Intercept *b***		(17.278, 61.199)	
Correlation			
r		0.12671	
r^2		0.016056	
t		1.4897	
***p* (uncorr.)**		0.13861	
**permutation *p***		0.1394	

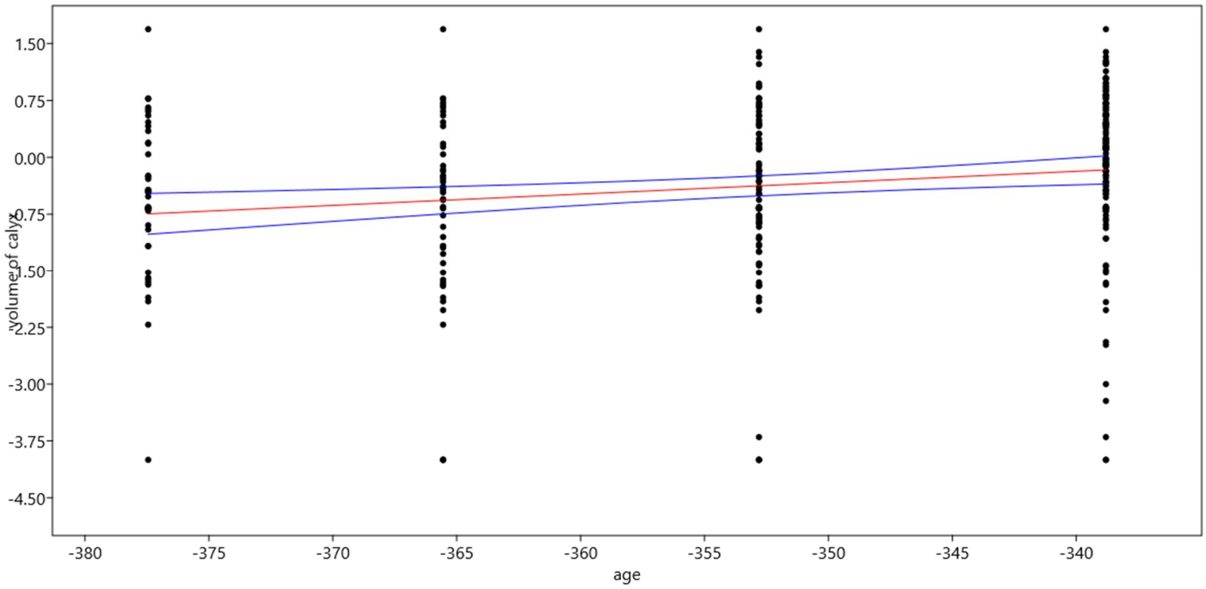

Supplementary Fig 18. OLS regression: Late Devonian-Early Carboniferous Log volume/age. [holotypes of type species of Pentacrinoidea binned to 4 stages in which given genera occurred using a "range through approach")].

Supplementary Table 24. OLS regression statistics: Late Devonian-Early Carboniferous sizes and age. [holotypes of type species of Pentacrinoidea binned to 4 stages in which given genera occurred using a "range through approach")]. Confidence intervals from 1999 bootstrapped replicates. Body volume transformed by Log_{10}.

**Slope *a***	0.01498	Std. error *a*	0.0048262
**Intercept *b***	4.9088	Std. error *b*	1.7033
95% bootstrapped confidence intervals (N=1999)			
**Slope *a***		(0.0056851, 0.024094)	
**Intercept *b***		(1.5918, 8.1007)	
Correlation			
r		0.17933	
***r*²**		0.03216	
t		3.1041	
***p* (uncorr.)**		0.0021	
**permutation *p***		0.002	

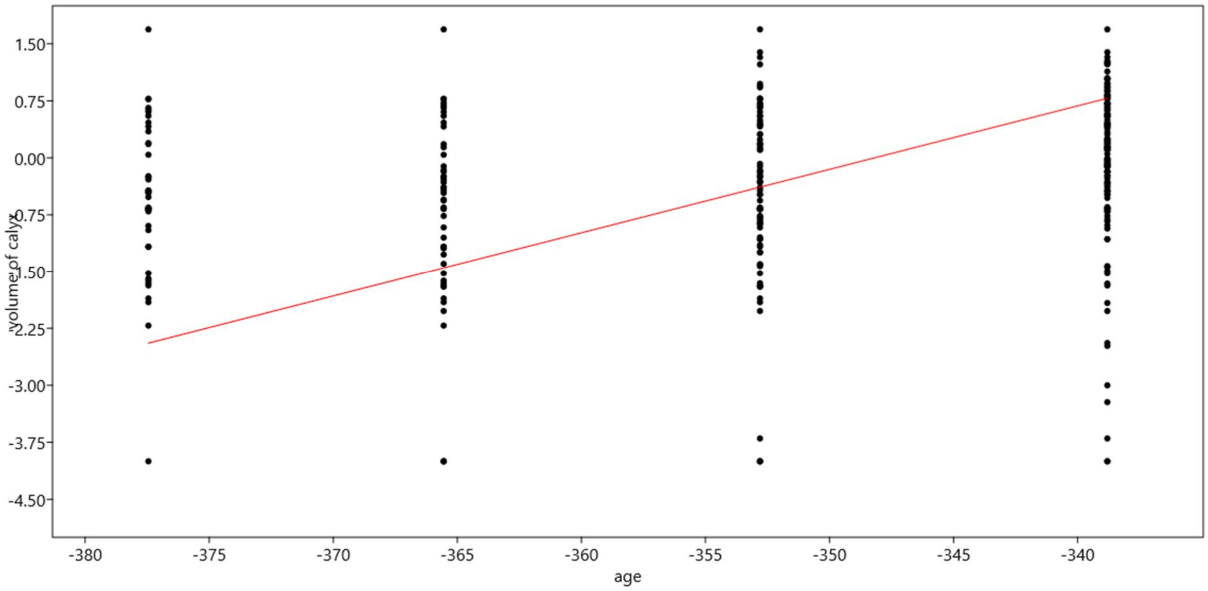

Supplementary Fig 19. RMA regression: Late Devonian-Early Carboniferous Log volume/age. [holotypes of type species of Pentacrinoidea binned to 4 stages in which given genera occurred using a "range through approach")].

Supplementary Table 25. RMA regression statistics: Late Devonian-Early Carboniferous sizes and age. [holotypes of type species of Pentacrinoidea binned to 4 stages in which given genera occurred using a "range through approach")]. Confidence intervals from 1999 bootstrapped replicates. Body volume transformed by Log_{10}.

**Slope *a***	0.08354	Std. error *a*	0.0048262
**Intercept *b***	29.087	Std. error *b*	2.9043
95% bootstrapped confidence intervals (N=1999)			
**Slope *a***		(0.072493, 0.093534)	
**Intercept *b***		(25.293, 32.504)	
Correlation			
r		0.17933	
r^2		0.03216	
t		3.1041	
***p* (uncorr.)**		0.0021	
**permutation *p***		0.0021	

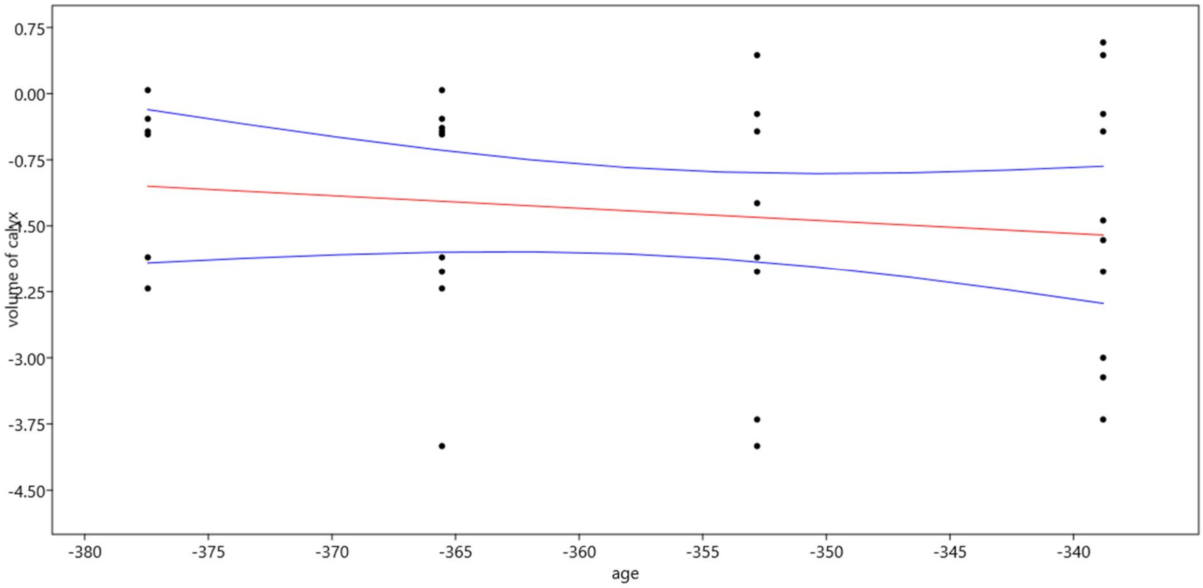

Supplementary Fig 20. OLS regression: Late Devonian-Early Carboniferous Log volume/age. [holotypes of type species of disparids binned to 4 stages in which given genera occurred using a "range through approach")].

Supplementary Table 26. OLS regression statistics: Late Devonian-Early Carboniferous sizes and age. [holotypes of type species of disparids binned to 4 stages in which given genera occurred using a "range through approach")]. Confidence intervals from 1999 bootstrapped replicates. Body volume transformed by Log_{10}.

**Slope *a***	-0.014312	Std. error *a*	0.01687
**Intercept *b***	-6.4524	Std. error *b*	6.0191
95% bootstrapped confidence intervals (N=1999)			
**Slope *a***		(-0.045549, 0.017944)	
**Intercept *b***		(-17.586, 5.0763)	
Correlation			
r		-0.15063	
***r*²**		0.02269	
t		-0.84836	
***p* (uncorr.)**		0.40274	
**permutation *p***		0.4088	

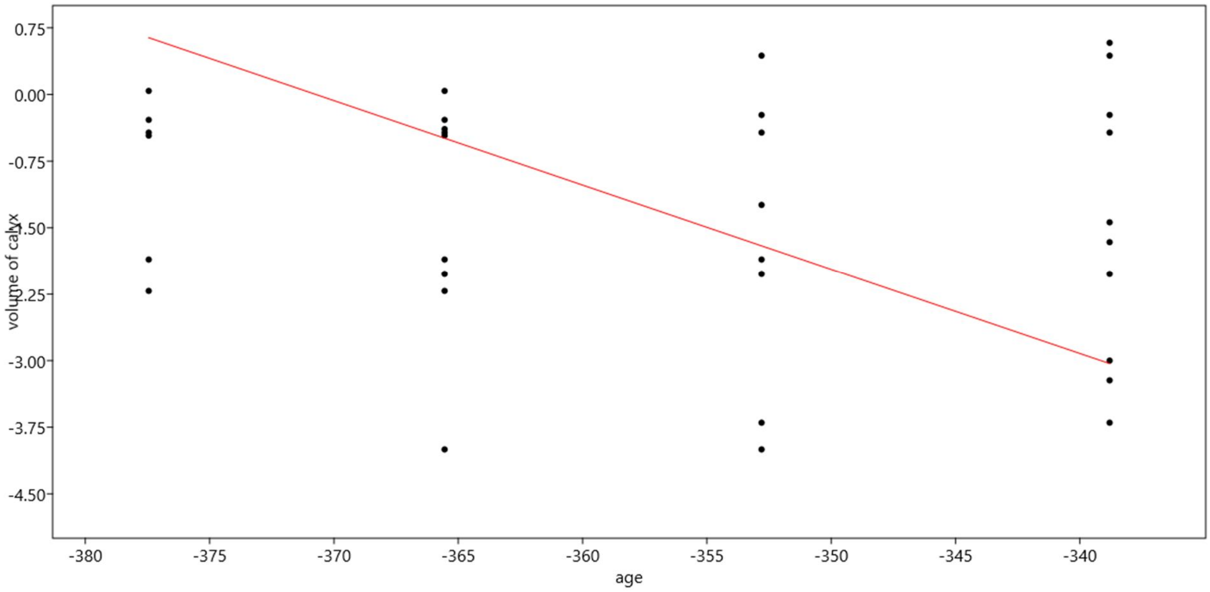

Supplementary Fig 21. RMA regression: Late Devonian-Early Carboniferous Log volume/age. [holotypes of type species of disparids binned to 4 stages in which given genera occurred using a "range through approach")].

Supplementary Table 27. RMA regression statistics: Late Devonian-Early Carboniferous sizes and age. [holotypes of type species of disparids binned to 4 stages in which given genera occurred using a "range through approach")]. Confidence intervals from 1999 bootstrapped replicates. Body volume transformed by Log_{10}.

**Slope *a***	-0.095011	Std. error *a*	0.01687
**Intercept *b***	-35.223	Std. error *b*	36.269
95% bootstrapped confidence intervals (N=1999)			
**Slope *a***	(-0.29857, -0.071233)		
**Intercept *b***	(-107.61, -26.423)		
Correlation			
r	-0.15063		
r^2	0.02269		
t	-0.84836		
***p* (uncorr.)**	0.40274		
**permutation *p***	0.407		

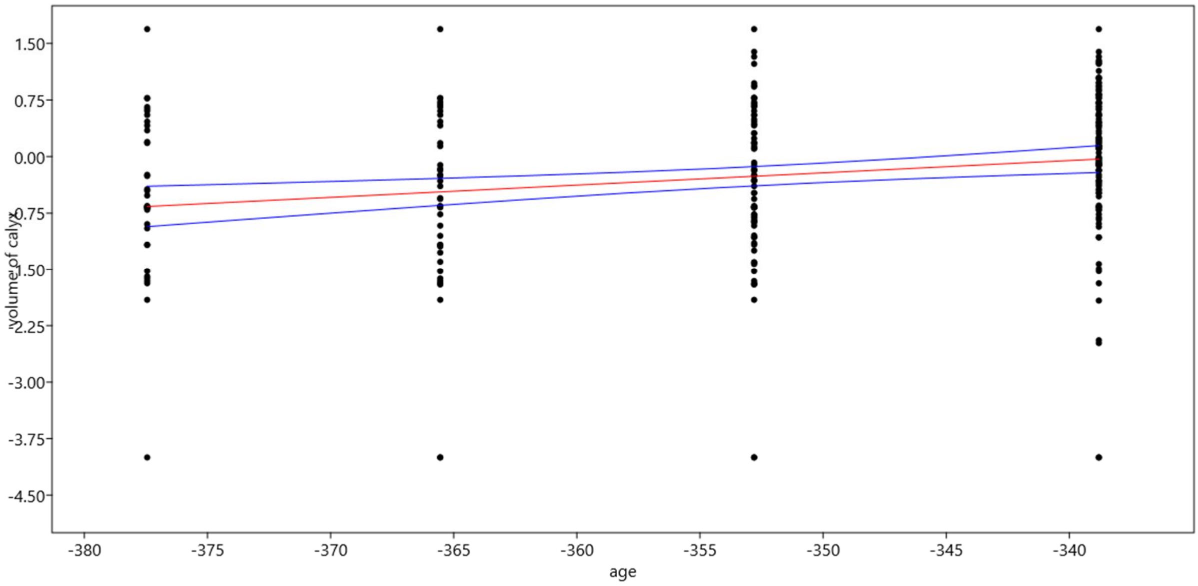

Supplementary Fig 22. OLS regression: Late Devonian-Early Carboniferous Log volume/age. [holotypes of type species of cladids binned to 4 stages in which given genera occurred using a "range through approach")].

Supplementary Table 28. OLS regression statistics: Late Devonian-Early Carboniferous sizes and age. [holotypes of type species of cladids binned to 4 stages in which given genera occurred using a "range through approach")]. Confidence intervals from 1999 bootstrapped replicates. Body volume transformed by Log_{10}.

**Slope *a***	0.016266	Std. error *a*	0.0047422
**Intercept *b***	5.4783	Std. error *b*	1.6713
95% bootstrapped confidence intervals (N=1999)			
**Slope *a***		(0.0069746, 0.025095)	
**Intercept *b***		(2.1542, 8.5927)	
Correlation			
r		0.20923	
r^2		0.043776	
t		3.4301	
***p* (uncorr.)**		0.00070282	
**permutation *p***		0.0008	

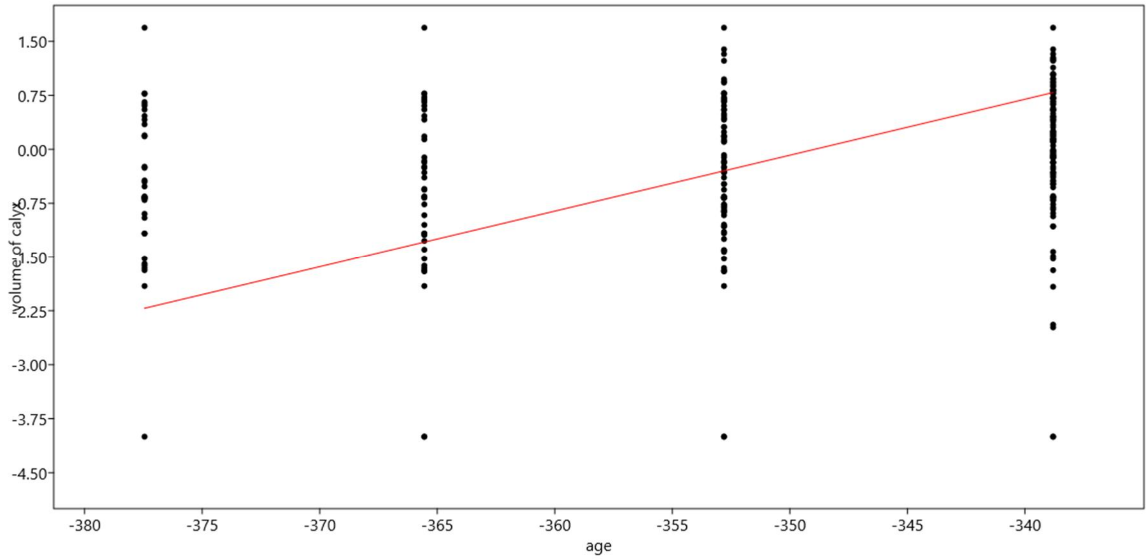

Supplementary Fig 23. RMA regression: Late Devonian-Early Carboniferous Log volume/age. [holotypes of type species of cladids binned to 4 stages in which given genera occurred using a "range through approach")].

Supplementary Table 29. RMA regression statistics: Late Devonian-Early Carboniferous sizes and age. [holotypes of type species of cladids binned to 4 stages in which given genera occurred using a "range through approach")]. Confidence intervals from 1999 bootstrapped replicates. Body volume transformed by Log_{10}.

**Slope *a***	0.077744	Std. error *a*	0.0047422
**Intercept *b***	27.129	Std. error *b*	2.796
95% bootstrapped confidence intervals (N=1999)			
**Slope *a***		(0.066109, 0.088737)	
**Intercept *b***		(23.101, 30.917)	
Correlation			
r		0.20923	
***r*²**		0.043776	
t		3.4301	
***p* (uncorr.)**		0.00070282	
**permutation *p***		0.0009	

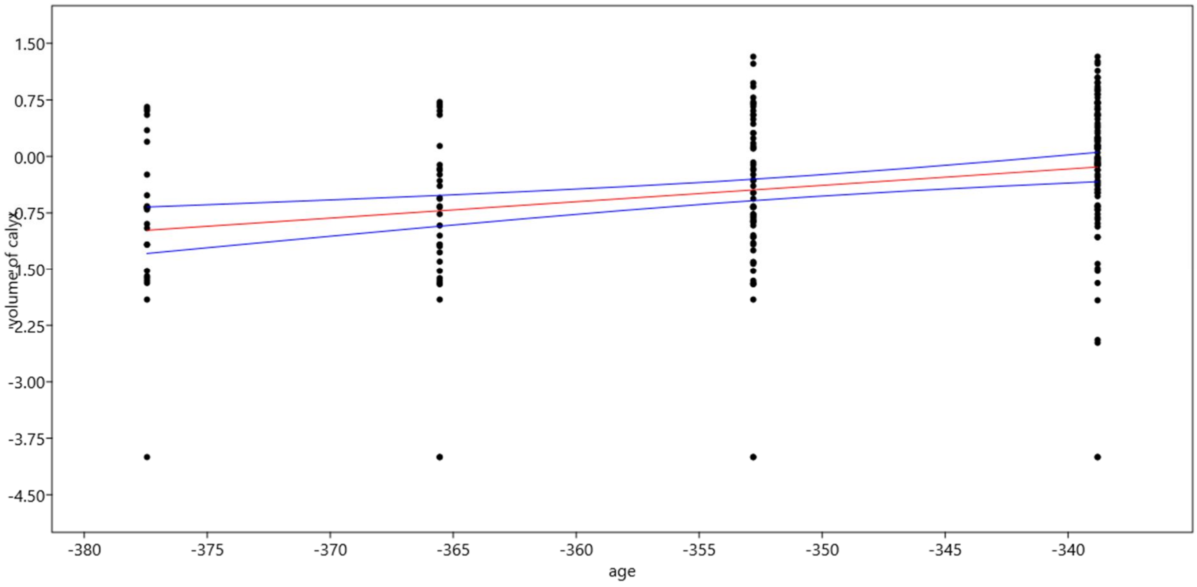

Supplementary Fig 24. OLS regression: Late Devonian-Early Carboniferous Log volume/age. [holotypes of type species of eucladids binned to 4 stages in which given genera occurred using a "range through approach")].

Supplementary Table 30. OLS regression statistics: Late Devonian-Early Carboniferous sizes and age. [holotypes of type species of eucladids binned to 4 stages in which given genera occurred using a "range through approach")]. Confidence intervals from 1999 bootstrapped replicates. Body volume transformed by Log_{10}.

**Slope *a***	0.02171	Std. error *a*	0.0053557
**Intercept *b***	7.2158	Std. error *b*	1.8838
95% bootstrapped confidence intervals (N=1999)			
**Slope *a***		(0.011369, 0.032023)	
**Intercept *b***		(3.605, 10.812)	
Correlation			
r		0.27123	
r^2		0.07357	
t		4.0544	
***p* (uncorr.)**		7.12E-05	
**permutation *p***		0.0001	

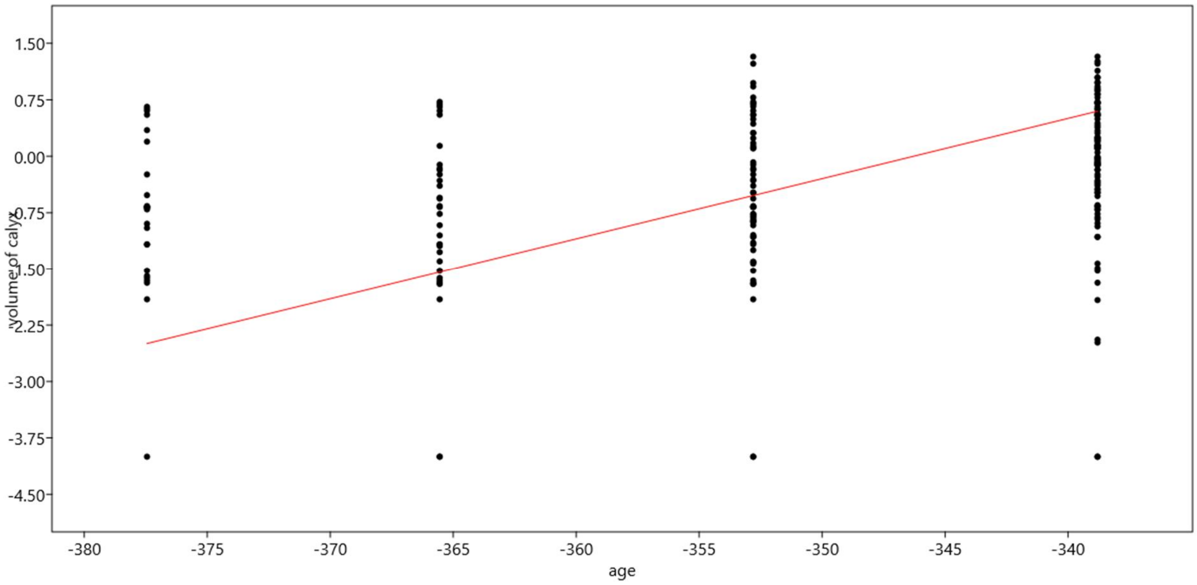

Supplementary Fig 25. RMA regression: Late Devonian-Early Carboniferous Log volume/age. [holotypes of type species of eucladids binned to 4 stages in which given genera occurred using a “range through approach”)].

Supplementary Table 31. RMA regression statistics: Late Devonian-Early Carboniferous sizes and age. [holotypes of type species of eucladids binned to 4 stages in which given genera occurred using a “range through approach”)]. Confidence intervals from 1999 bootstrapped replicates. Body volume transformed by Log_{10}.

**Slope *a***	0.08006	Std. error *a*	0.0053557
**Intercept *b***	27.722	Std. error *b*	3.5516
95% bootstrapped confidence intervals (N=1999)			
**Slope *a***		(0.066927, 0.092146)	
**Intercept *b***		(23.262, 31.901)	
Correlation			
r		0.27123	
r^2		0.07357	
t		4.0544	
***p* (uncorr.)**		7.12E-05	
**permutation *p***		0.0001	

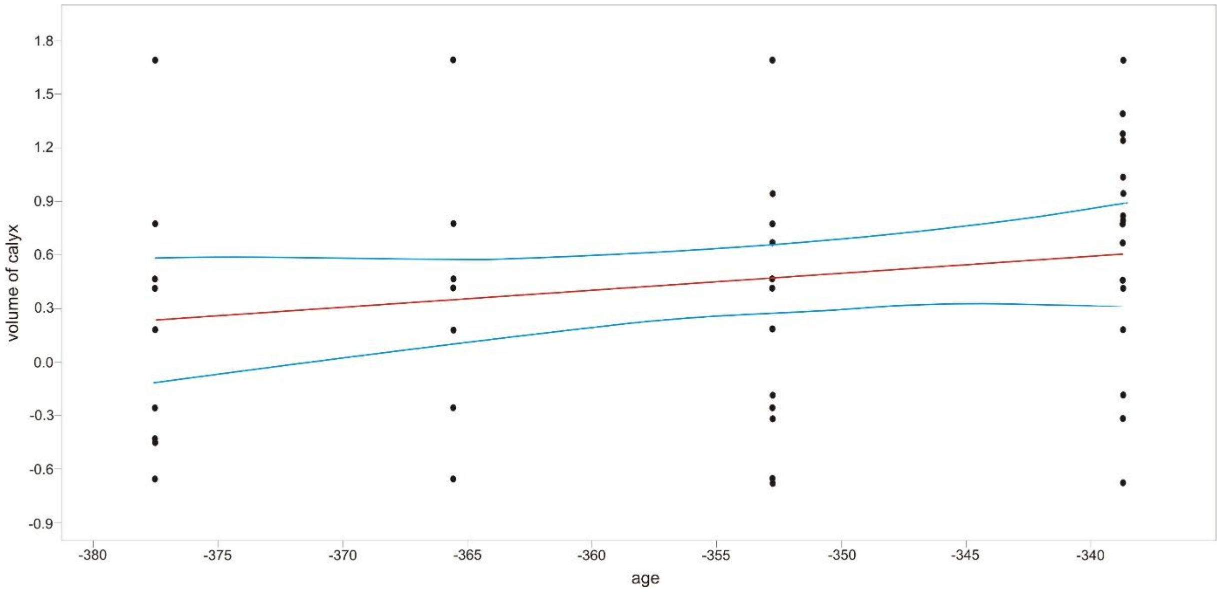

Supplementary Fig 26. OLS regression: Late Devonian-Early Carboniferous Log volume/age. [holotypes of type species of flexibles binned to 4 stages in which given genera occurred using a "range through approach")].

Supplementary Table 32. OLS regression statistics: Late Devonian-Early Carboniferous sizes and age. [holotypes of type species of flexibles binned to 4 stages in which given genera occurred using a "range through approach")]. Confidence intervals from 1999 bootstrapped replicates. Body volume transformed by Log_{10}.

**Slope *a***	0.0093414	Std. error *a*	0.0065986
**Intercept *b***	3.7609	Std. error *b*	2.3445
95% bootstrapped confidence intervals (N=1999)			
**Slope *a***		(-0.0029071, 0.023064)	
**Intercept *b***		(-0.57234, 8.6067)	
Correlation			
r		0.2002	
***r*²**		0.040079	
t		1.4157	
***p* (uncorr.)**		0.16333	
**permutation *p***		0.1704	

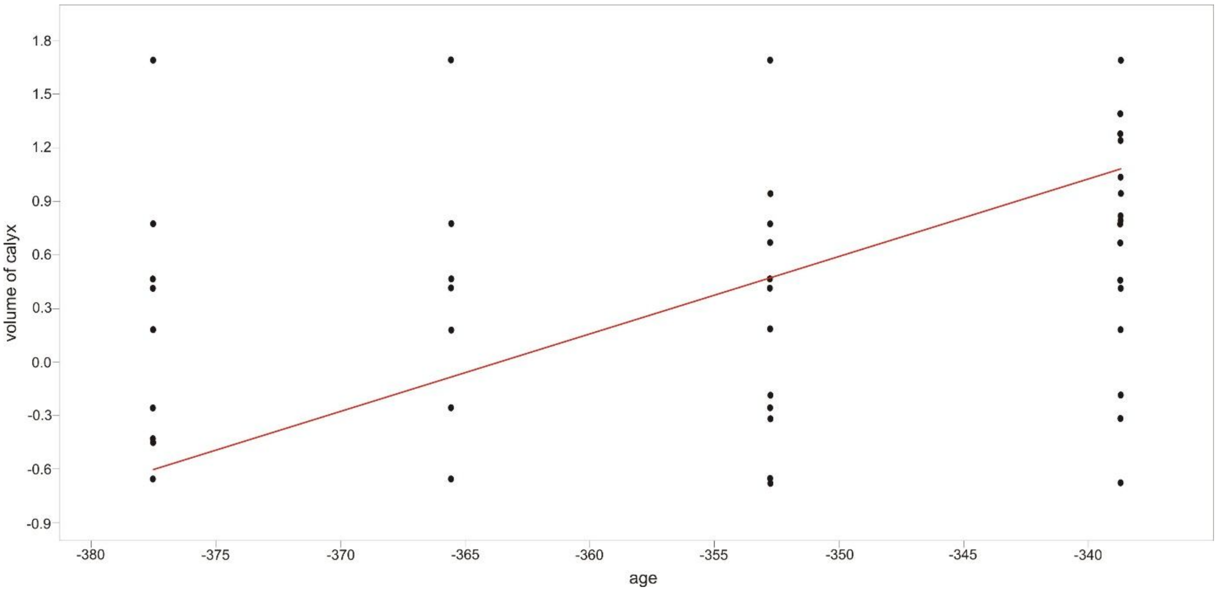

Supplementary Fig 27. RMA regression: Late Devonian-Early Carboniferous Log volume/age. [holotypes of type species of flexibles binned to 4 stages in which given genera occurred using a "range through approach")].

Supplementary Table 33. RMA regression statistics: Late Devonian-Early Carboniferous sizes and age. [holotypes of type species of flexibles binned to 4 stages in which given genera occurred using a "range through approach")]. Confidence intervals from 1999 bootstrapped replicates. Body volume transformed by Log_{10}.

**Slope *a***	0.046661	Std. error *a*	0.0065986
**Intercept *b***	17.01	Std. error *b*	5.5025
95% bootstrapped confidence intervals (N=1999)			
**Slope *a***		(0.036963, 0.1434)	
**Intercept *b***		(13.669, 51.303)	
Correlation			
r		0.2002	
r^2		0.040079	
t		1.4157	
***p* (uncorr.)**		0.16333	
**permutation *p***		0.1658	

Supplementary Table 34. Database comprising estimated volumes of calyces of analyzed crinoid genera.

No.	Genus	Genus range	Type species	Type species age	Estimated volume
	**Cl. Crinoidea**				
	**Subclass Camerata**				
	**Or. Diplobathrida**				
**1**	*Cribanocrinus*	Tournaisian-Visean	*Rhodocrinus wortheni*	Tournaisian	2.7902
**2**	*Gilbertsocrinus*	Givetian-Visean	*Gilbertsocrinus calcaratus*	Tournaisian	7.4129
**3**	*Pterinocrinus*	Emsian-Frasnian	*Pterinocrinus quinquenodus*	Frasnian	0.2508
**4**	*Rhipidocrinus*	Eifelian-Famennian	*Rhodocrinites crenatus*	Givetian	9.0582
**5**	*Rhodocrinites*	Pragian-Serpukhovian	*Rhodocrinites verus*	Tournaisian	2.9821
	**Or. Monobathrida**				
**6**	*Aacocrinus*	Tournaisian-Bashkirian	*Aacocrinus nododorsatus*	Tournaisian	3.6024
**7**	*Abactinocrinus*	Famennian-Tournaisian	*Abactinocrinus rossei*	Tournaisian	3.2495
**8**	*Abatocrinus*	Tournaisian-Visean	*Actinocrinus turbinatus*	Tournaisian	5.8605
**9**	*Acrocrinus*	Visean-Serpukhovian	*Acrocrinus shumardi*	Visean	30.1075
**10**	*Actinocrinites*	Tournaisian-Artinskian	*Actinocrinites triacontadactylus*	Tournaisian	16.7059
**11**	*Agaricocrinus*	Tournaisian-Visean	*Agaricocrinus tuberosus*	Tournaisian-Visean	19.8313
**12**	*Agathocrinus*	Emsian-Famennian	*Agathocrinus globosus*	Frasnian-Famennian	0.9903
**13**	*Alloprosallocrinus*	Visean	*Alloprosallocrinus conicus*	Visean	5.2840
**14**	*Amphoracrinus*	Tournaisian-Visean	*Actinocrinus gilbertsoni*	Tournaisian	21.5273
**15**	*Amphoracrocrinus*	Visean-Serpukhovian	*Acrocrinus amphora*	Serpukhovian	1.9965
**16**	*Ancalocrinus*	Tournaisian	*Actinocrinus spinobrachiatus*	Tournaisian	4.7383
**17**	*Aorocrinus*	Givetian-Visean	*Dorycrinus immaturus*	Tournaisian	1.0500
**18**	*Arthroacantha*	Emsian-Frasnian	*Arthroacantha ithacensis*	Frasnian	1.5635
**19**	*Aryballocrinus*	Tournaisian-Visean	*Actinocrinus (Megistocrinus) whitei*	Tournaisian	32.1204
**20**	*Athabascacrinus*	Famennian-Tournaisian	*Athabascacrinus colemanensis*	Tournaisian	3.1745
**21**	*Azygocrinus*	Tournaisian	*Actinocrinus dodecadactylus*	Tournaisian	1.8027

**22**	*Batocrinus*	Visean	*Batocrinus icosidactylus*	Visean	11.9863
**23**	*Blairocrinus*	Tournaisian	*Blairocrinus trijugis*	Tournaisian	1.4003
**24**	*Brahmacrinus*	Tournaisian-Visean	*Brahmacrinus ponderosus*	Tournaisian	16.2723
**25**	*Cactocrinus*	Tournaisian	*Actinocrinus proboscidialis*	Tournaisian	10.2774
**26**	*Camptocrinus*	Visean-Artinskian	*Camptocrinus myelodactylus*	Visean	0.0244
**27**	*Cantharocrinus*	Emsian-Famennian	*Cantharocrinus minor*	Emsian	0.3124
**28**	*Cerasmocrinus*	Frasnian	*Cerasmocrinus springeri*	Frasnian	5.0667
**29**	*Chinacrinus*	Famennian	*Chinacrinus xinjiangensis*	Famennian	0.3548
**30**	*Coelocrinus*	Tournaisian	*Actinocrinus concavus*	Tournaisian	0.4236
**31**	*Ctenocrinus*	Wenlock-Frasnian	*Ctenocrinus typus*	Ludlovian	20.1838
**32**	*Cusacrinus*	Tournaisian	*Actinocrinus nodobrachiatus*	Tournaisian	4.2129
**33**	*Cytidocrinus*	Tournaisian	*Actinocrinus sculptus*	Tournaisian	154.1682
**34**	*Dialutocrinus*	Tournaisian	*Dialutocrinus milleri*	Tournaisian	7.7995
**35**	*Dichocrinus*	Tournaisian-Wordian	*Dichocrinus radiatus*	Tournaisian	3.3929
**36**	*Dilatocrinus*	Tournaisian	*Amphoracrinus multiramosus*	Tournaisian	8.5870
**37**	*Dizygocrinus*	Tournaisian-Visean	*Actinocrinus indianaensis*	Visean	2.8927
**38**	*Dorycrinus*	Tournaisian-Visean	*Dorycrinus mississippiensis*	Visean	26.7778
**39**	*Ectocrinus*	Tournaisian-Serpukhovian	*Actinocrinus olla*	Visean	38.7924
**40**	*Eretmocrinus*	Tournaisian-Moscovian	*Eretmocrinus magnificus*	Visean	12.0957
**41**	*Eumorphocrinus*	Tournaisian-Visean	*Eumorphocrinus erectus*	Visean	15.3659
**42**	*Eutrochocrinus*	Tournaisian-Visean	*Actinocrinus christyi*	Visean	23.7337
**43**	*Gennaeocrinus*	Givetian-Famennian	*Actinocrinus kentuckiensis*	Givetian	11.2246
**44**	*Glaphyrocrinus*	Tournaisian-Visean	*Glaphyrocrinus expansus*	Visean	29.6485
**45**	*Globocrinus*	Visean-Serpukhovian	*Batocrinus unionensis*	Visean	3.0979
**46**	*Hexacrinites*	Ludlovian-Tournaisian	*Platycrinus interscapularis*	Givetian	22.3290
**47**	*Hyrtanecrinus*	Visean-Serpukhovian	*Hyrtanecrinus diabolus*	Visean	0.2115
**48**	*Ilmocrinus*	Visean-Serpukhovian	*Ilmocrinus dissymmetricus*	Serpukhovian	24.3368
**49**	*Iotacrinus*	Tournaisian	*Iotacrinus dorsatus*	Tournaisian	4.4452
**50**	*Lenneocrinus*	Emsian-Frasnian	*Lenneocrinus cirratus*	Givetian	0.0586

**51**	*Macrocrinus*	Tournaisian-Visean	*Asterocrinus konincki*	Visean	1.4276
**52**	*Maligneocrinus*	Tournaisian	*Maligneocrinus medicinensis*	Tournaisian	25.4825
**53**	*Manillocrinus*	Tournaisian	*Cactocrinus? brownei*	Tournaisian	5.0393
**54**	*Megistocrinus*	Emsian-Tournaisian	*Actinocrinus evansii*	Tournaisian	76.2013
**55**	*Melocrinites*	Wenlock-Visean	*Melocrinites hieroglyphicus*	Frasnian	2.2533
**56**	*Nunnacrinus*	Tournaisian-Serpukhovian	*Nunnacrinus mamillatus*	Tournaisian-Visean	6.5237
**57**	*Oenochoacrinus*	Emsian-Tournaisian	*Oenochoacrinus princeps*	Emsian	0.8752
**58**	*Paradichocrinus*	Tournaisian-Visean	*Paradichocrinus polydactylus*	Tournaisian-Visean	19.2837
**59**	*Physetocrinus*	Famennian-Bashkirian	*Actinocrinus ventricosus*	Tournaisian-Visean	21.6388
**60**	*Pimlicocrinus*	Tournaisian-Bashkirian	*Amphoracrinus clitheroensis*	Visean	12.7790
**61**	*Platycrinites*	Eifelian-Guadalupian	*Platycrinites laevis*	Tournaisian	1.8067
**62**	*Plemnocrinus*	Tournaisian	*Plemnocrinus beebei*	Tournaisian	7.9039
**63**	*Pleurocrinus*	Tournaisian-Guadalupian	*Platycrinites mucronatus*	Tournaisian	6.2180
**64**	*Protacrocrinus*	Tournaisian	*Acrocrinus primitivus*	Tournaisian	0.0690
**65**	*Pterotocrinus*	Visean-Serpukhovian	*Asterocrinus capitalis*	Serpukhovian	1.8389
**66**	*Sampsonocrinus*	Tournaisian-Bashkirian	*Sampsonocrinus hemisphericus*	Tournaisian	7.7948
**67**	*Springeracrocrinus*	Tournaisian-Moscovian	*Acrocrinus intermedius*	Visean	0.0578
**68**	*Steganocrinus*	Tournaisian	*Actinocrinus pentagonaus*	Tournaisian	5.5500
**69**	*Stomiocrinus*	Tournaisian-Artinskian	*Stomiocrinus subglobosus*	Artinskian	0.2504
**70**	*Strimplecrinus*	Famennian-Serpukhovian	*Dichocrinus plicatus*	Tournaisian	0.3520
**71**	*Strotocrinus*	Tournaisian	*Actinocrinus perumbrosus*	Tournaisian	6.1115
**72**	*Sunwaptacrinus*	Tournaisian	*Sunwaptacrinus brazeauensis*	Tournaisian	8.3825
**73**	*Talarocrinus*	Visean-Serpukhovian	*Dichocrinus cornigerus*	Visean	0.8288
**74**	*Tarantocrinus*	Tournaisian	*Tarantocrinus typus*	Tournaisian	18.0363
**75**	*Teleiocrinus*	Tournaisian-Visean	*Actinocrinus umbrosus*	Tournaisian-Visean	34.0335
**76**	*Thinocrinus*	Tournaisian-Artinskian	*Thinocrinus westheadi*	Tournaisian	6.8823
**77**	*Trichotocrinus*	Frasnian	*Melocrinus harrisi*	Frasnian	0.1765
**78**	*Uperocrinus*	Famennian-Visean	*Actinocrinus pyriformis*	Tournaisian	16.0133
**79**	*Wacrinus*	Famennian	*Wacrinus caseyensis*	Famennian	6.9408

	**Parvclass Disparida**				
**80**	*Allagecrinus*	Famennian-Serpukhovian	*Allagecrinus austinii*	Tournaisian	0.0095
**81**	*Allocatillocrinus*	Visean-Artinskian	*Allagecrinus carpenteri*	Visean	0.0218
**82**	*Anamesocrinus*	Givetian-Famennian	*Anamesocrinus lutheri*	Givetian	0.0061
**83**	*Belemnocrinus*	Tournaisian-Visean	*Belemnocrinus typus*	Tournaisian	0.5886
**84**	*Calycanthocrinus*	Emsian-Famennian	*Calycanthocrinus decadactylus*	Emsian	1.0971
**85**	*Catillocrinus*	Tournaisian-Bashkirian	*Catillocrinus tennesseeae*	Tournaisian	2.7340
**86**	*Desmacriocrinus*	Famennian-Tournaisian	*Kallimorphocrinus weldenensis*	Tournaisian	0.0001
**87**	*Eucatillocrinus*	Visean	*Catillocrinus bradleyi*	Visean	3.8110
**88**	*Halysiocrinus*	Pragian-Visean	*Cheirocrinus dactylus*	Tournaisian	0.3728
**89**	*Haplocrinites*	Ludlovian-Tournaisian	*Haplocrinites sphaeroideus*	Eifelian	0.0139
**90**	*Jaekelicrinus*	Frasnian-Famennian	*Jaekelicrinus bashkiricus*	Eifelian	0.3449
**91**	*Kallimorphocrinus*	Visean-Wordian	*Kallimorphocrinus astrus typicus*	Baskhirian	0.0006
**92**	*Isoallagecrinus*	Visean-Wordian	*Allagecrinus bassleri*	Baskhirian	0.0365
**93**	*Playfordicrinus*	Famennian	*Playfordicrinus kellyensis*	Famennian	0.4082
**94**	*Storthingocrinus*	Eifelian-Famennian	*Platycrinus fritillus*	Eifelian	0.5185
**95**	*Thaminocrinus*	Visean	*Allagecrinus biplex*	Visean	0.0010
**96**	*Trophocrinus*	Tournaisian-Bashkirian	*Trophocrinus tumidus*	Tournaisian	0.0002
**97**	*Whiteocrinus*	Tournaisian	*Belemnocrinus florifer*	Tournaisian	0.0573
	**Parvclass Cladida**				
	**Primitive cladids**				
**98**	*Abrachiocrinus*	Visean-Artinskian	*Sycocrinites clausus*	Visean	0.0322
**99**	*Amphipsalidocrinus*	Givetian-Artinskian	*Amphipsalidocrinus scissurus*	Bashkirian	0.0001
**100**	*Aulodesocrinus*	Visean	*Aulodesocrinus parvus*	Visean	0.0033
**101**	*Barycrinus*	Tournaisian-Visean	*Cyathocrinus spurius*	Tournaisian-Visean	17.0077
**102**	*Belanskicrinus*	Frasnian	*Bactrocrinus westoni*	Frasnian	0.1116
**103**	*Carlopsocrinus*	Visean	*Carlopsocrinus bullatus*	Visean	0.0036
**104**	*Cestocrinus*	Visean	*Cestocrinus striatus*	Visean	1.6204

**105**	*Clistocrinus*	Givetian-Artinskian	*Clistocrinus pyriformis*	Bashkirian	0.0300
**106**	*Codiacrinus*	Lochkovian-Frasnian	*Codiacrinus granulatus*	Eifelian	2.2238
**107**	*Costalocrinus*	Pragian-Visean	*Poteriocrinus dilatatus*	Eifelian	4.0252
**108**	*Cradeocrinus*	Givetian-Tournaisian	*Cradeocrinus elongatus*	Frasnian	0.0221
**109**	*Cyathocrinites*	Wenlock-Wordian	*Cyathocrinites planus*	Tournaisian	3.5631
**110**	*Cydonocrinus*	Visean-Artinskian	*Cydonocrinus parvulus*	Visean	0.1289
**111**	*Dichostreblocrinus*	Tournaisian-Artinskian	*Dichostreblocrinus scrobiculus*	Bashkirian	1.2980
**112**	*Edapocrinus*	Visean	*Edapocrinus rugosus*	Visean	7.3358
**113**	*Fiannacrinus*	Tournaisian	*Poteriocrinus quinquangularis*	Tournaisian	0.0899
**114**	*Goniocrinus*	Eifelian-Visean	*Goniocrinus sculptilis*	Tournaisian	0.0206
**115**	*Iteacrinus*	Emsian-Frasnian	*Iteacrinus flagellum*	Frasnian	0.2122
**116**	*Lageniocrinus*	Visean-Artinskian	*Lageniocrinus seminulum*	Visean	0.1980
**117**	*Lampadosocrinus*	Tournaisian-Sakmarian	*Dichostreblocrinus minutus*	Tournaisian-Visean	0.0001
**118**	*Pagecrinus*	Emsian-Frasnian	*Pagecrinus gracilis*	Emsian	0.0254
**119**	*Parabotryocrinus*	Frasnian	*Parabotryocrinus tschudovensis*	Frasnian	0.2065
**120**	*Parisocrinus*	Eifelian-Visean	*Poteriocrinites perplexus*	Tournaisian	0.0677
**121**	*Pellecrinus*	Tournaisian-Visean	*Cyathocrinus hexadactylus*	Tournaisian	5.0724
**122**	*Quantoxocrinus*	Givetian-Famennian	*Quantoxocrinus ussheri*	Givetian	0.0673
**123**	*Saccosompsis*	Visean	*Cyathocrinus insperatus*	Visean	0.2180
**124**	*Sycocrinites*	Visean	*Sycocrinites anapeptamenus*	Visean	0.4528
**125**	*Zygiosocrinus*	Tournaisian	*Zygiosocrinus typicus*	Tournaisian	0.0124
**126**	*Zygotocrinus*	Tournaisian	*Zygotocrinus fragilis*	Tournaisian	0.1333
	**Advanced cladids**				
**127**	*Abrotocrinus*	Tournaisian-Visean	*Abrotocrinus cymosus*	Tournaisian	3.5378
**128**	*Acylocrinus*	Tournaisian-Visean	*Acylocrinus tumidus*	Tournaisian	0.3278
**129**	*Adiakritocrinus*	Tournaisian	*Adiakritocrinus oviatti*	Tournaisian	0.0200
**130**	*Adinocrinus*	Visean	*Zeacrinus nodosus*	Visean	18.1652
**131**	*Aenigmocrinus*	Visean	*Poteriocrinus anomalus*	Visean	0.0121
**132**	*Agassizocrinus*	Visean-Serpukhovian	*Agassizocrinus conicus*	Visean	3.4578

**133**	*Amabilicrinus*	Famennian-Tournaisian	*Amabilicrinus iranensis*	Tournaisian	0.4049
**134**	*Amadeusicrinus*	Famennian	*Pachylocrinus subpentagonalis*	Famennian	0.0642
**135**	*Ampelocrinus*	Visean-Serpukhovian	*Ampelocrinus bernhardinae*	Visean	0.3580
**136**	*Anartiocrinus*	Visean-Serpukhovian	*Anartiocrinus lyoni*	Visean	1.3722
**137**	*Anemetocrinus*	Visean	*Anemetocrinus biserialis*	Visean	0.9419
**138**	*Aphelecrinus*	Famennian-Serpukhovian	*Aphelecrinus elegans*	Visean	0.1717
**139**	*Apokryphocrinus*	Tournaisian	*Apokryphocrinus wellsvillensis*	Tournaisian	0.0391
**140**	*Armenocrinus*	Visean	*Armenocrinus watersi*	Visean	0.8972
**141**	*Ascetocrinus*	Tournaisian	*Scaphiocrinus rusticellus*	Tournaisian	0.1691
**142**	*Aulocrinus*	Visean	*Aulocrinus agassizi*	Visean	2.4130
**143**	*Blothrocrinus*	Famennian-Visean	*Poteriocrinus jesupi*	Tournaisian	4.8913
**144**	*Bollandocrinus*	Tournaisian-Visean	*Poteriocrinus conicus*	Tournaisian	4.5801
**145**	*Borucrinus*	Tournaisian-Visean	*Dinotocrinus eirensis*	Tournaisian	0.0375
**146**	*Bridgerocrinus*	Famennian-Tournaisian	*Bridgerocrinus fairyensis*	Tournaisian	0.0198
**147**	*Bronaughocrinus*	Visean-Serpukhovian	*Bronaughocrinus figuratus*	Visean	7.9765
**148**	*Bufalocrinus*	Famennian	*Catactocrinus? torus*	Famennian	0.0890
**149**	*Bursacrinus*	Tournaisian	*Bursacrinus wachsmuthi*	Tournaisian	2.0485
**150**	*Catactocrinus*	Frasnian-Famennian	*Catactocrinus leptodactylus*	Frasnian	0.0239
**151**	*Cercidocrinus*	Tournaisian	*Poteriocrinus bursaeformis*	Tournaisian	2.7024
**152**	*Charientocrinus*	Givetian-Frasnian	*Charientocrinus ithacensis*	Frasnian	0.1269
**153**	*Coeliocrinus*	Tournaisian	*Poteriocrinus dilatatus*	Tournaisian	0.1600
**154**	*Corematocrinus*	Frasnian	*Corematocrinus plumosus*	Frasnian	0.1971
**155**	*Corythocrinus*	Visean	*Corythocrinus romingeri*	Visean	0.8787
**156**	*Cosmetocrinus*	Famennian-Serpukhovian	*Cosmetocrinus gracilis*	Tournaisian	0.7731
**157**	*Cromyocrinus*	Visean-Moscovian	*Cromyocrinus simplex*	Osagean	4.2901
**158**	*Culmicrinus*	Tournaisian-Serpukhovian	*Poteriocrinus regularis*	Visean	0.1556
**159**	*Cupressocrinites*	Pragian-Famennian	*Cupressocrinites crassus*	Givetian	4.5436
**160**	*Cydrocrinus*	Tournaisian-Visean	*Poteriocrinus coxanus*	Tournaisian	6.0469
**161**	*Cymbiocrinus*	Visean-Bashkirian	*Cymbiocrinus romingeri*	Visean	0.2245

**162**	*Dasciocrinus*	Visean-Serpukhovian	*Cyathocrinus florialis*	Visean	0.4301
**163**	*Decadocrinus*	Givetian-Visean	*Poteriocrinus scalaris*	Tournaisian	0.5734
**164**	*Delocrinus*	Visean-Artinskian	*Poteriocrinus hemisphericus*	Visean	2.1887
**165**	*Derbiocrinus*	Visean	*Derbiocrinus diversus*	Visean	6.7166
**166**	*Derorhethocrinus*	Tournaisian	*Derorhethocrinus elongatus*	Tournaisian	0.0398
**167**	*Dinotocrinus*	Visean	*Dinotocrinus compactus*	Visean	0.2970
**168**	*Eratocrinus*	Tournaisian-Visean	*Zeacrinus elegans*	Tournaisian	2.0358
**169**	*Eupachycrinus*	Visean-Serpukhovian	*Graphiocrinus quatuordecimbrachialis*	Visean	3.7371
**170**	*Exaetocrinus*	Visean-Stephanian	*Stuartwellercrinus argentinei*	Moscovian	0.6607
**171**	*Exochocrinus*	Visean-Serpukhovian	*Eupachycrinus tumulosus*	Visean	6.6278
**172**	*Fifeocrinus*	Visean-Serpukhovian	*Pachylocrinus tielensis*	Visean	11.1499
**173**	*Forthocrinus*	Visean	*Forthocrinus lepidus*	Visean	1.7414
**174**	*Gaelicrinus*	Tournaisian	*Poteriocrinus rostratus*	Tournaisian	0.1379
**175**	*Gelasinocrinus*	Tournaisian	*Gelasinocrinus revimentus*	Tournaisian	0.2745
**176**	*Gilmocrinus*	Tournaisian-Visean	*Gilmocrinus iowensis*	Tournaisian	0.3311
**177**	*Glossocrinus*	Frasnian-Famennian	*Glossocrinus naplesensis*	Frasnian-Famennian	0.0124
**178**	*Goleocrinus*	Visean-Moscovian	*Goleocrinus masonensis*	Bashkirian	1.8029
**179**	*Graphiocrinus*	Tournaisian-Wordian	*Graphiocrinus encrinoides*	Tournaisian	0.0851
**180**	*Grabauicrinus*	Famennian	*"Decadocrinus" xinjiangensis*	Famennian	0.2740
**181**	*Hallocrinus*	Emsian-Frasnian	*Cyathocrinus ornatissimus*	Frasnian	1.5643
**182**	*Heliosocrinus*	Visean-Bashkirian	*Heliosocrinus aftonensis*	Visean	11.1285
**183**	*Histocrinus*	Tournaisian-Visean	*Poteriocrinus (Scytalocrinus) grandis*	Tournaisian	1.2695
**184**	*Holcocrinus*	Famennian-Visean	*Graphiocrinus longicirrifer*	Tournaisian	0.6874
**185**	*Hosieocrinus*	Visean	*Tribrachiocrinus caledonicus*	Visean	0.8135
**186**	*Hutkocrinus*	Tournaisian	*Hutkocrinus kermanensis*	Tournaisian	0.0712
**187**	*Hydreionocrinus*	Visean-Serpukhovian	*Hydreionocrinus woodianus*	Visean	1.5405
**188**	*Hylodecrinus*	Visean	*Hylodecrinus sculptus*	Visean	3.1345
**189**	*Hypselocrinus*	Famennian-Serpukhovian	*Poteriocrinus hoveyi*	Tournaisian	1.3753
**190**	*Idosocrinus*	Visean	*Idosocrinus bispinosus*	Visean	0.7622

**191**	*Julieticrinus*	Famennian	*Julieticrinus romeo*	Famennian	0.2814
**192**	*Lanecrinus*	Tournaisian-Moscovian	*Scaphiocrinus depressus*	Tournaisian-Visean	0.2129
**193**	*Lebetocrinus*	Tournaisian-Visean	*Lebetocrinus grandis*	Tournaisian	8.4611
**194**	*Lekocrinus*	Visean	*Scaphiocrinus divaricatus*	Visean	0.5341
**195**	*Linobrachiocrinus*	Frasnian	*Linocrinus kindlei*	Frasnian	0.3048
**196**	*Linocrinus*	Tournaisian-Serpukhovian	*Linocrinus wachsmuthi*	Visean	0.1463
**197**	*Liparocrinus*	Famennian	*Liparocrinus batheri*	Famennian	0.0533
**198**	*Logocrinus*	Givetian-Tournaisian	*Logocrinus geniculatus*	Givetian	0.2115
**199**	*Lophocrinus*	Visean	*Lophocrinus speciosus*	Visean	0.1947
**200**	*Lorocrinus*	Tournaisian	*Lorocrinus zanguensis*	Tournaisian	0.4891
**201**	*Maevecrinus*	Tournaisian	*Maevecrinus bothros*	Tournaisian	1.4956
**202**	*Mantikosocrinus*	Visean-Serpukhovian	*Mantikosocrinus castus*	Visean	0.8148
**203**	*Maragnicrinus*	Frasnian	*Maragnicrinus portlandicus*	Frasnian	4.2773
**204**	*Meniscocrinus*	Visean	*Meniscocrinus magnitubus*	Visean	5.0633
**205**	*Nactocrinus*	Tournaisian	*Nactocrinus nitidus*	Tournaisian	0.2112
**206**	*Nudalocrinus*	Tournaisian	*Culmicrinus jeffersonensis*	Tournaisian	3.1136
**207**	*Ophiurocrinus*	Tournaisian-Moscovian	*Poteriocrinus originarius*	Moscovian	1.7384
**208**	*Pachylocrinus*	Famennian-Serpukhovian	*Scaphiocrinus aequalis*	Tournaisian	0.4754
**209**	*Paracosmetocrinus*	Tournaisian	*Paracosmetocrinus straki*	Tournaisian	0.1389
**210**	*Parascytalocrinus*	Visean	*Scytalocrinus validus*	Visean	0.9562
**211**	*Parazeacrinites*	Visean	*Zeacrinus konincki*	Visean	4.1884
**212**	*Pedinocrinus*	Visean	*Pachylocrinus clavatus*	Visean	5.2105
**213**	*Pelecocrinus*	Tournaisian-Serpukhovian	*Pelecocrinus insignis*	Tournaisian	9.4083
**214**	*Pentaramicrinus*	Visean-Serpukhovian	*Cromyocrinus gracilis*	Visean	0.4077
**215**	*Pentececrinus*	Famennian	*Pentececrinus parvus*	Famennian	0.0001
**216**	*Phacelocrinus*	Tournaisian-Bashkirian	*Poteriocrinus wetherbyi*	Tournaisian-Visean	0.8328
**217**	*Phanocrinus*	Visean-Serpukhovian	*Zeacrinus formosus*	Visean	1.3767
**218**	*Plaxocrinus*	Visean-Sakmarian	*Hydreionocrinus crassidiscus*	Moscovian	1.1237
**219**	*Poteriocrinites*	Famennian-Visean	*Poteriocrinites crassus*	Tournaisian-Visean	5.2815

**220**	*Prininocrinus*	Frasnian-Tournaisian	*Prininocrinus robustus*	Frasnian	0.2180
**221**	*Proampelocrinus*	Tournaisian	*Proampelocrinus himalayaensis*	Tournaisian	0.0566
**222**	*Ramulocrinus*	Tournaisian-Serpukhovian	*Ramulocrinus nigelensis*	Tournaisian	0.0848
**223**	*Rhabdocrinus*	Visean-Serpukhovian	*Poteriocrinus scotocarbonarius*	Visean	9.6363
**224**	*Rhopocrinus*	Visean-Serpukhovian	*Rhopocrinus spinosus*	Visean	1.6404
**225**	*Sarocrinus*	Visean	*Sarocrinus nitidus*	Visean	0.3695
**226**	*Scotiacrinus*	Visean-Serpukhovian	*Pachylocrinus tyriensis*	Visean	7.6341
**227**	*Scytalocrinus*	Famennian-Stephanian	*Scaphiocrinus robustus*	Tournaisian	0.6598
**228**	*Snowycrinus*	Visean	*Snowycrinus stonehouseensis*	Visean	0.1172
**229**	*Sostronocrinus*	Famennian-Tournaisian	*Sostronocrinus superbus*	Tournaisian	0.1213
**230**	*Springericrinus*	Tournaisian-Visean	*Poteriocrinus magniventrus*	Tournaisian	21.0803
**231**	*Stinocrinus*	Visean	*Stinocrinus granulosus*	Visean	1.2767
**232**	*Tarassocrinus*	Famennian	*Tarassocrinus synchlydus*	Famennian	0.0401
**233**	*Tholocrinus*	Visean-Serpukhovian	*Hydreionocrinus spinosus*	Serpukhovian	0.7663
**234**	*Tyrieocrinus*	Visean-Serpukhovian	*Tyrieocrinus laxus*	Visean	2.5711
**235**	*Ulrichicrinus*	Visean-Moscovian	*Ulrichicrinus oklahoma*	Serpukhovian	13.6795
**236**	*Ureocrinus*	Visean-Serpukhovian	*Poteriocrinus bockschii*	Visean	0.9864
**237**	*Worthenocrinus*	Visean	*Worthenocrinus patenus*	Visean	1.4273
**238**	*Zeacrinites*	Visean-Serpukhovian	*Zeacrinites magnoliaeformis*	Visean	2.7498
	**Superorder Flexibilia**				
	**Or. Taxocrinida**				
**239**	*Enascocrinus*	Visean	*Talanterocrinus redesdalensis*	Visean	2.8879
**240**	*Eutaxocrinus*	Wenlock-Tournaisian	*Taxocrinus affinis*	Eifelian	0.2212
**241**	*Meristocrinus*	Wenlock-Tournaisian	*Taxocrinus (Gnorimocrinus) loveni*	Gotland	0.5539
**242**	*Onychocrinus*	Tournaisian-Serpukhovian	*Onychocrinus exsculptus*	Tournaisian	24.5446
**243**	*Parichthyocrinus*	Tournaisian-Visean	*Ichthyocrinus nobilis*	Tournaisian	0.4826
**244**	*Taxocrinus*	Eifelian-Serpukhovian	*Cyathocrinus macrodactylus*	Frasnian-Tournaisian	1.5132
	**Or. Sagenocrinida**				
**245**	*Aexitrophocrinus*	Visean-Stephanian	*Synerocrinus formosus*	Serpukhovian	6.6049

**246**	*Ainacrinus*	Tournaisian-Visean	*Synerocrinus? smithi*	Visean	0.2105
**247**	*Amphicrinus*	Visean-Moscovian	*Amphicrinus scoticus*	Visean	17.3476
**248**	*Ancoracrinus*	Tournaisian	*Ancoracrinus typus*	Tournaisian	1.5418
**249**	*Apodactylocrinus*	Frasnian	*Apodactylocrinus keithi*	Frasnian	0.3698
**250**	*Artichthyocrinus*	Visean-Wolffian	*Artichthyocrinus springeri*	Visean	2.8545
**251**	*Caldenocrinus*	Visean	*Caldenocrinus curtus*	Visean	6.2785
**252**	*Clidochirus*	Ashgillian-Visean	*Clidochirus pyrum*	Wenlock	5.9020
**253**	*Dactylocrinus*	Eifelian-Tournaisian	*Dimerocrinites oligoptilus*	Frasnian	2.9095
**254**	*Dieuryocrinus*	Visean	*Euryocrinus duplex*	Visean	10.7582
**255**	*Euryocrinus*	Givetian-Visean	*Euryocrinus concavus*	Tournaisian	5.9969
**256**	*Forbesiocrinus*	Famennian-Visean	*Forbesiocrinus nobilis*	Tournaisian	49.0625
**257**	*Gaulocrinus*	Visean	*Stemmatocrinus trautscholdi*	Visean	18.8166
**258**	*Mespilocrinus*	Tournaisian-Visean	*Mespilocrinus forbesianus*	Tournaisian	0.6513
**259**	*Metichthyocrinus*	Tournaisian-Visean	*Ichthyocrinus burlingtonensis*	Tournaisian	8.7677
**260**	*Nipterocrinus*	Tournaisian-Visean	*Nipterocrinus wachsmuthi*	Tournaisian	4.6547
**261**	*Synaptocrinus*	Givetian-Famennian	*Forbesiocrinus nuntius*	Givetian	0.3524
**262**	*Wachsmuthicrinus*	Frasnian-Visean	*Forbesiocrinus thiemei*	Tournaisian	2.5837

References

1. Borths, M. R. & Ausich, W. I. Ordovician-Silurian Lilliput crinoids during the end-Ordovician biotic crisis. *Swiss J. Palaeontol.* **130**, 7–18 (2011).

2. Moore, R. C. & Teichert, C. Treatise on Invertebrate Paleontology. Part T, Echinodermata 2, 403–812 (Geological Society of America and University of Kansas Press, 1978).

3. Kirk, E. *Zygotocrinus*, a new fossil inadunate crinoid genus. *Am. Jour. Sci.* **242**, 190–203 (1943).

4. Strimple, H. L. & Levorson, C. O. Additional Crinoid Specimens from the Shellrock Formation (Upper Devonian) of Iowa. *Proceedings of the Iowa Academy of Science* **80**, 182–184 (1973).

5. Broadhead, T. W. & Strimple, H. L. *Hyrtanecrinus*, a new Carboniferous camerate crinoid genus from Eastern North America. *J. Paleontol.* **54**, 35–44 (1980).

6. Broadhead, T. W. Carboniferous camerate crinoid subfamily Dichocrininae. *Palaeontogr. Abt. A* **176**, 81–157 (1981).

7. Solovjeva, M. V. A new genus of camerate crinoid from the Carboniferous of the Urals. *Paleontol. J.* **4**, 114–118 (1984).

8. Ausich, W. I. Early Silurian Inadunate crinoids (Brassfield Formation, Ohio). *J. Paleontol.* **60**, 719–735 (1986).

9. Webster, G. D. & Lane, N. G. Crinoids from the Anchor Limestone (Lower Mississippian) of the Monte Cristo Group, Southern Nevada. *The University of Kansas Paleontological Contributions* **119**, 1–55 (1987).

10. Lindley, I. D. *Glaphyrocrinus*, a new camerate crinoid genus from Lower Carboniferous of New South Wales. *Alcheringa* **12**, 129–136 (1988).

11. Kammer, T. W. & Ausich, W. I. Advanced cladid crinoids from the Middle Mississippian of the East-Central United States: intermediate-grade cycles. *J. Paleontol.* **67**, 614–639 (1993).

12. Kammer, T. W. & Ausich, W. I. Advanced cladid crinoids from the Middle Mississippian of the East-Central United States: advanced-grade cycles. *J. Paleontol.* **68**, 339–351 (1994).

13. Kammer, T. W. & Ausich, W. I. Primitive cladid crinoids from Upper Osagean-Lower Meramecian (Mississippian) rocks of the East-Central United States. *J. Paleontol.* **70**, 835–866 (1996).

14. Jell, P. A. Silurian and Devonian crinoids from Central Vistoria. *Mem. Queensl. Mus.* **43**, 1–114 (1999).

15. Webster, G. D. Lower Carboniferous echinoderms from Northern Utah and Western Wyoming. *Paleontology Series, Volume 1* **128**, 1–65 (1997).
16. Webster, G. D. & Jell, P. A. New Carboniferous crinoids from eastern Australia. *Mem. Queensl. Mus.* **43**, 237–278 (1999).
17. Webster, G. D., Hafley, D. J., Blake, D. B. & Glass, A. Crinoids and stelleroids (Echinodermata) from the Broken Rib Member, Dyer Formation (Late Devonian, Famennian) of the White River Plateau, Colorado. *J. Paleontol.* **73**, 461–486 (1999).
18. Webster, G. D., Maples, C. G., Mawson, R. & Dastanpour, M. A cladid-dominated early Mississippian crinoid and conodont fauna from Kerman Province, Iran and revisionof the glossocrinids and rhenocrinids. *J. Paleontol.* **77**, 1–36, http://dx.doi.org/10.1666/0022-3360(2003)77[1:ACEMCA]2.0.CO;2 (2003).
19. Ausich, W. I. & Sevastopulo, G. D. The Lower Carboniferous (Tournaisian) crinoids from Hook Head, County Wexford, Ireland. *Monograph of the Palaeontolographical Society*, 1–136 (2001).
20. Waters, J. A. *et al.* A quadrupling of Fammenian Pelmatozoan diversity: new Late Devonian blastoids and crinoids from Northwest China. *J. Paleontol.* **77**, 922–948, http://dx.doi.org/10.1666/0022-3360(2003)077<0922:AQOFPD>2.0.CO;2 (2003).
21. Webster, G. D. & Waters, J. A. Early Chesterian Echinoderms from the Otter Formation of Central Montana. *The Mountain Geologist* **42**, 23–34 (2004).
22. Ausich, W. I. & Kammer, T. W. Generic concepts in the Amphoracrinidae Bather, 1899 (Class Crinoidea) and evaluation of generic assignments of North American species. *J. Paleontol.* **82**, 1139–1149 (2008).
23. Rhenberg, E. C. & Kammer, T. W. Camerate Crinoids from the Nunn Member (Tournaisian, Osagean) of the Lower Mississippian Lake Valley Formation, New Mexico. *J. Paleontol.* **87**, 312–340, https://doi:10.1666/12-033R.1 (2013).
24. Segessenman, D. C. & Kammer, T. W. Testing reduced evolutionary rates during the Late Palaeozoic Ice Age using the crinoid fossil record. *Lethaia*, https://doi:10.1111/let.12239 (2017).
25. Heim, N. A., Knope, M. L., Schaal, E. K., Wang, S. C. & Payne, J. L. Cope's rule in the evolution of marine animals. *Science* **347**, 867–870, https://doi:10.1126/science.1260065 (2015).
26. Dommergues, J. L., Montuire, S. & Neige, P. Size patterns through time: The case of the Early Jurassic ammonite radiation. *Paleobiology* **28**, 423–434, https://doi:10.1666/0094-8373(2002)028<0423:SPTTTC>2.0.CO;2 (2002).

27. Payne, J. L. Evolutionary dynamics of gastropod size across the end-Permian extinction and through the Triassic recovery interval. *Paleobiology* **31**, 269–290, https://doi:10.1666/0094-8373(2005)031[0269:EDOGSA]2.0.CO;2 (2005).
28. Thompson, J. R. & Ausich, W. I. Facies distribution and taphonomy of echinoids from the Fort Payne Formation (late osagean, early Visean, Mississippian) of Kentucky. *J. Paleontol.* **90**, 239–249, https://doi:10.1017/jpa.2016.46 (2015).
29. Brom, K. R., Salamon, M. A., Ferré, B., Brachaniec, T. & Szopa, K. The Lilliput effect in crinoids at the end of the Oceanic Anoxic Event 2: a case study from Poland. *J. Paleontol.* **89**, 1076–1081, https://doi:10.1017/jpa.2016.10 (2015).
30. Kosnik, M. A., Jablonski, D., Lockwood, R. & Novack-Gottshall, P. M. Quantifying molluscan body size in evolutionary and ecological analyses: Maximizing the return on datacollection efforts. *Palaios* **21**: 588–597, https://doi:10.2110/palo.2006.p06-012r (2006).
31. Krause, R. A., Stempien, J. A., Kowalewski, M. J. & Miller, A. I. Body size estimates from the literature: Utility and potential for macroevolutionary studies. *Palaios* **22**: 60–73, https://doi:10.2110/palo.2005.p05-122r (2007).
32. Hammer, Ø., Harper, D. A. T. & Ryan, P. D. PAST: Paleontological statistics software package for education and data analysis. *Palaeontol. Electron.* **4**, 1–9 (2001).
